# Supplementary material for: A subset of megakaryocytes regulates development of hematopoietic stem cell precursors
Source: EMBO J. 2024 Apr 5;43(9):1722–39. doi: 10.1038/s44318-024-00079-4 (PMC11065989; doi:10.1038/s44318-024-00079-4)
Supplement: Supplementary file 1 — Appendix [file 44318_2024_79_MOESM1_ESM.pdf]

A subset of Megakaryocytes Regulates the Development of Hematopoietic Stem Cell Precursors via secreting Tnfsf14

Wenlang Lan[1, †], Jinping Li[1, †], Zehua Ye[1, †], Yumin Liu[1], Sifan Luo[1], Xun Lu[1], Zhan Cao[1], Yifan Chen[1], Hongtian Chen[1], Zhuan Li[1, *]

*Corresponding author. Zhuan Li, Email: zhuanli2018@smu.edu.cn

Appendix Figures

Figure Name	Page
**Appendix Figure S1**: Analyzing hematopoietic related cells in the PF4-Cre;Rosa-tdTomato embryos.	3-5
**Appendix Figure S2**: Hematopoietic related cells in the PF4-Cre;Rosa-DTA (DTA) yolk sac and fetal liver.	6-7
**Appendix Figure S3**: The pre-Hematopoietic stem cells were unchanged in the E10.5 PF4-Cre;Rosa-DTA (DTA) AGM region.	8-10
**Appendix Figure S4**: Single-cell RNA-sequencing analysis identifying the differences between distinct subfractions of megakaryocytes and flow analysis confirmed the alteration of $CD226^+$ megakaryocytes in the DTA AGM regions.	11-15
**Appendix Figure S5**: Single-cell RNA-sequencing analysis supports the synergy of megakaryocytes and macrophages on the hematopoiesis in the embryos.	16-18
**Appendix Table S1**: Primers for genotype and qRT-PCR.	19

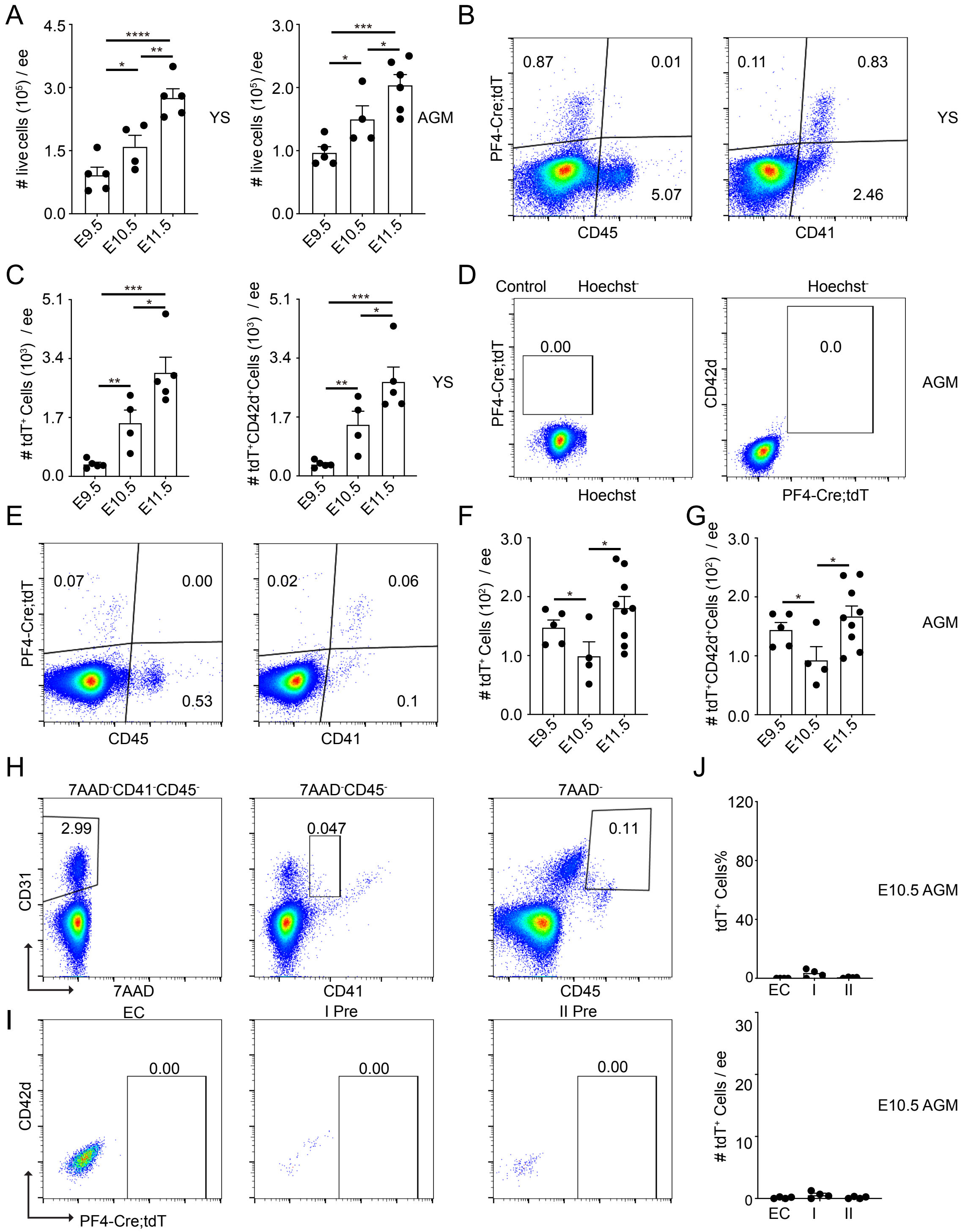
A
live cells (10^5) / ee
YS
live cells (10^5) /ee
AGM
E9.5
E10.5
E11.5
B
PF4-Cre;tdT
CD45
CD41
0.87
0.01
5.07
0.11
0.83
2.46
YS
C
tdT+ Cells (10^3) / ee
tdT+CD42d+Cells (10^3) / ee
YS
D
Control
Hoechst-
Hoechst
PF4-Cre;tdT
CD42d
0.00
0.0
AGM
E
0.07
0.00
0.53
0.02
0.06
0.1
F
tdT+ Cells (10^2) / ee
G
tdT+CD42d+Cells (10^2) / ee
AGM
H
7AAD-CD41-CD45-
7AAD-CD45-
7AAD-
CD31
7AAD
CD41
CD45
2.99
0.047
0.11
I
EC
I Pre
II Pre
CD42d
PF4-Cre;tdT
0.00
J
tdT+ Cells%
tdT+ Cells / ee
E10.5 AGM
EC
I
II

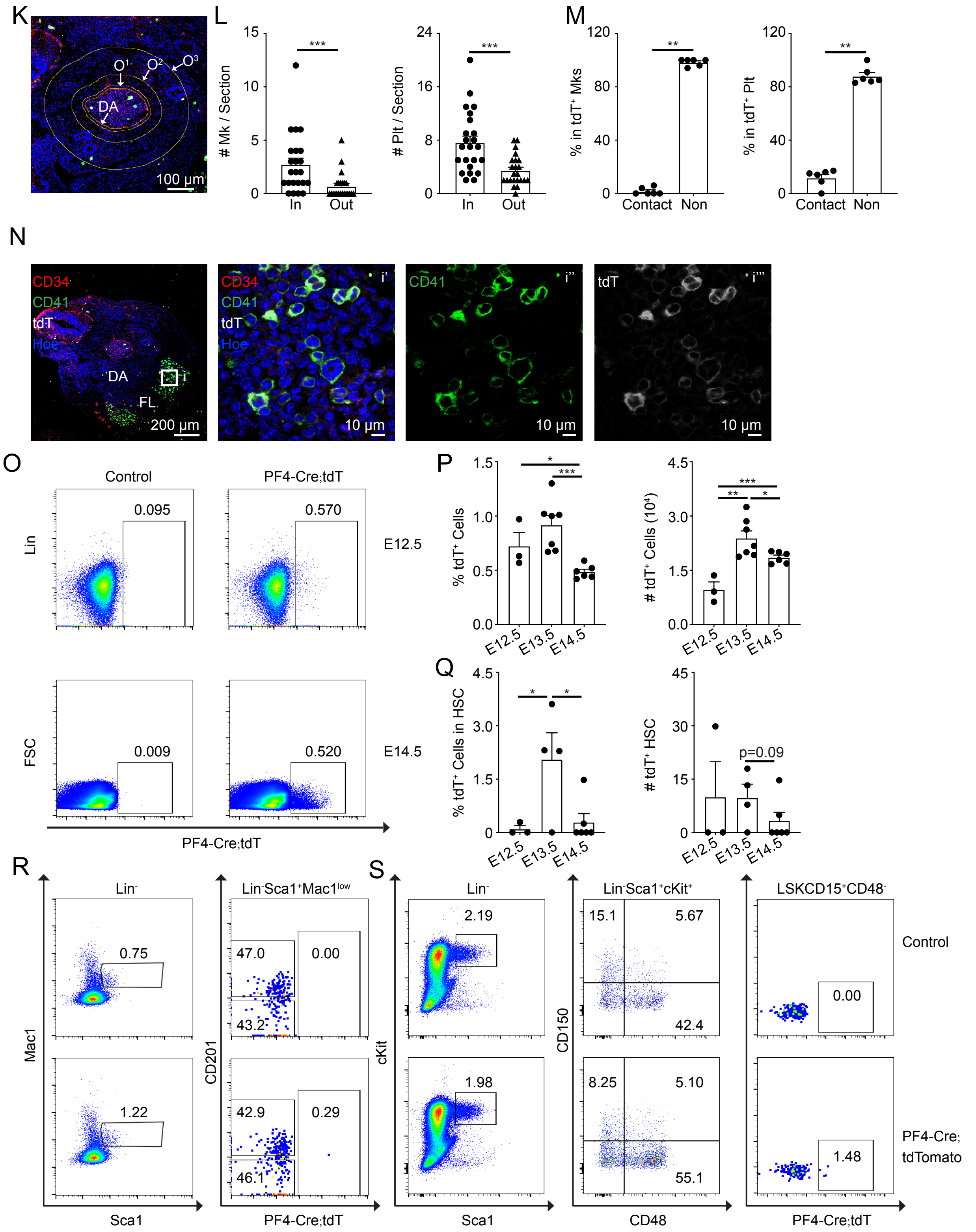

Appendix Figure S1 (Related to Fig.1) : Analyzing hematopoietic related cells in the PF4-Cre;Rosa-tdTomato embryos. (A) The number of live cell in the E9.5-E11.5 yolk

sac and AGM regions. n=4-5 fetuses from at least 3 litters/condition, *p<0.05, **p=0.0051, ***p=0.0002, ****p<0.0001. (B-G) Presentative flow cytometric analysis and the numbers of tdTomato^{+} cells or tdTomato^{+}CD42d^{+} cells showing the expression of CD45 and CD41 in PF4-Cre;Rosa-tdTomato^{+} cells in the E10.5 yolk sac (B-C) and AGM (D-G). n=4-9 fetuses from at least 3 litters/condition, *p<0.05, **p<0.01, ***p<0.001. (H-J) Flow cytometric analysis data displaying PF4 failed to label EC (CD31^{+}CD41^{-}CD45^{-}), pre-HSC I (CD31^{+}CD41^{low}CD45^{-}), and pre-HSC II (CD31^{+}CD45^{+}) in the E10.5 AGM region. n=4 fetuses from at least 3 litters/condition. (K) The area of strategy for counting megakaryocytes (Mks) and platelets in the immunostaining sections. O1=Area (10 μm) out of aorta, O2=Area (50 μm) out of aorta, O3=Area (150 μm) out of aorta. Red=CD34, Blue=Hoechst, Green=CD41, White=tdTomato (tdT). (L) The number of Mks and platelets per section in the dorsal aorta and out of the aorta (150 μm, including O1-O3 areas). n=23 sections of 3 fetuses from 3 litters. Statistical significance was determined by Mann-Whitney test. ***p<0.001. (M) Immunostaining data showing the percentage of Mks (82 cells) and platelets (255 cells) contacted or non-contacted with endothelial cells in the dorsal aorta. n=6 fetuses from at least 3 litters/condition. Statistical significance was determined by Mann-Whitney test. **p<0.01. Non=Non-contacted. (N) Immunostaining of cryosections in the E10.5 fetal liver. Scale Bar presents 200 μm or 10 μm. Red=CD34, Green=CD41, White=tdTomato (tdT), Blue=Hoechst. (O) Presentative flow cytometric analysis showing the percentage of tdTomato^{+} cells in live cells of E12.5 and E14.5 fetal liver. (P) The percentage and total cell number of tdTomato^{+} cells in E12.5-14.5 fetal liver. n=3-7 fetuses from at least 3 litters/condition, *p<0.05, **p=0.0016, ***p<0.001. (Q) The percentage of tdTomato^{+} cells in HSCs and cell number of tdTomato^{+} HSCs in E12.5-14.5 fetal liver. n=3-7 fetuses from at least 3 litters/condition, *p<0.05. (R-S) Presentative flow cytometric analysis tdTomato^{+} cells in HSCs of E12.5 and E14.5 fetal liver. E12.5 HSC defined by Lin^{-}Mac1^{low}Sca1^{+}CD201^{+} and E13.5-E14.5 HSC defined by Lin^{-}Scal^{+}cKit^{+}CD150^{+}CD48^{-}.

Data Information: For all analysis above bars represent mean±SEM. Statistical significance was determined by unpaired Student's t-test unless the statistical test was indicated.

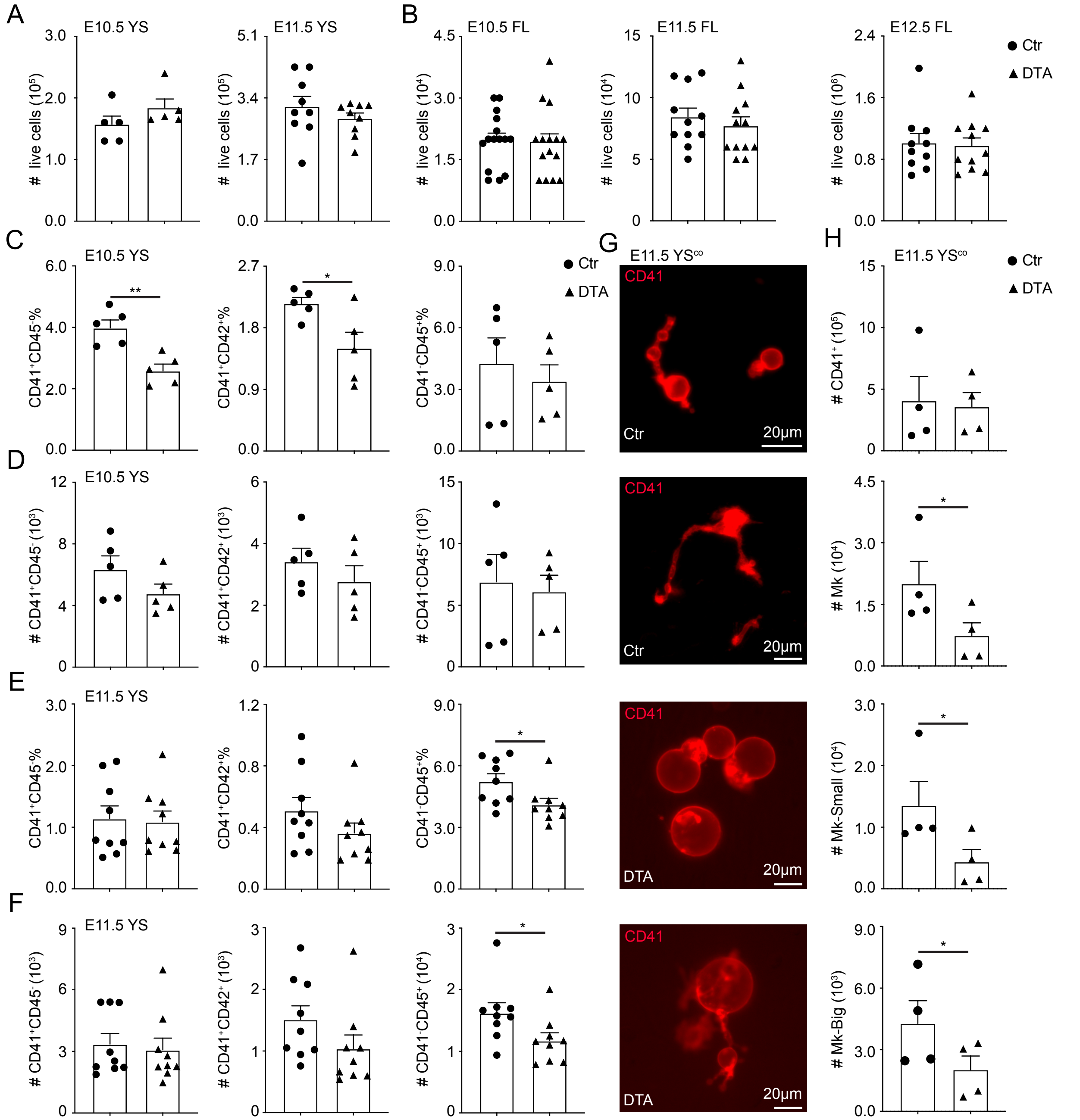

Appendix Figure S2 (Related to Fig. 2) : Hematopoietic related cells in the PF4-Cre;Rosa-DTA (DTA) yolk sac and fetal liver. (A-B) The number of live cells in the E10.5-E11.5 yolk sac(A) and E12.5 fetal liver(B). n=5-15 fetuses from at least 3 litters/condition. (C-F) Percentages and cell numbers of Mks and $CD45^+$ hematopoietic cells in the E10.5 and E11.5 yolk sac. n=5-9 fetuses from at least 3 litters/condition, *$p<0.05$, **$p<0.01$. (G) The morphology of $CD41^+$ Mks and platelets derived E11.5 control and DTA yolk sac after 3 days OP9-DL1 coculture. Red=CD41. Scale bar=20 μm. (H)

The reduction of total Mks ($CD41^+CD42d^+$) and bigger/smaller Mks derived PF4-Cre;DTA yolk sac compared with control. n=4 independent experiments, *$p<0.05$, YS = yolk sac.

Data Information: For all analysis above bars represent mean±SEM. Statistical significance was determined by unpaired Student's t-test.

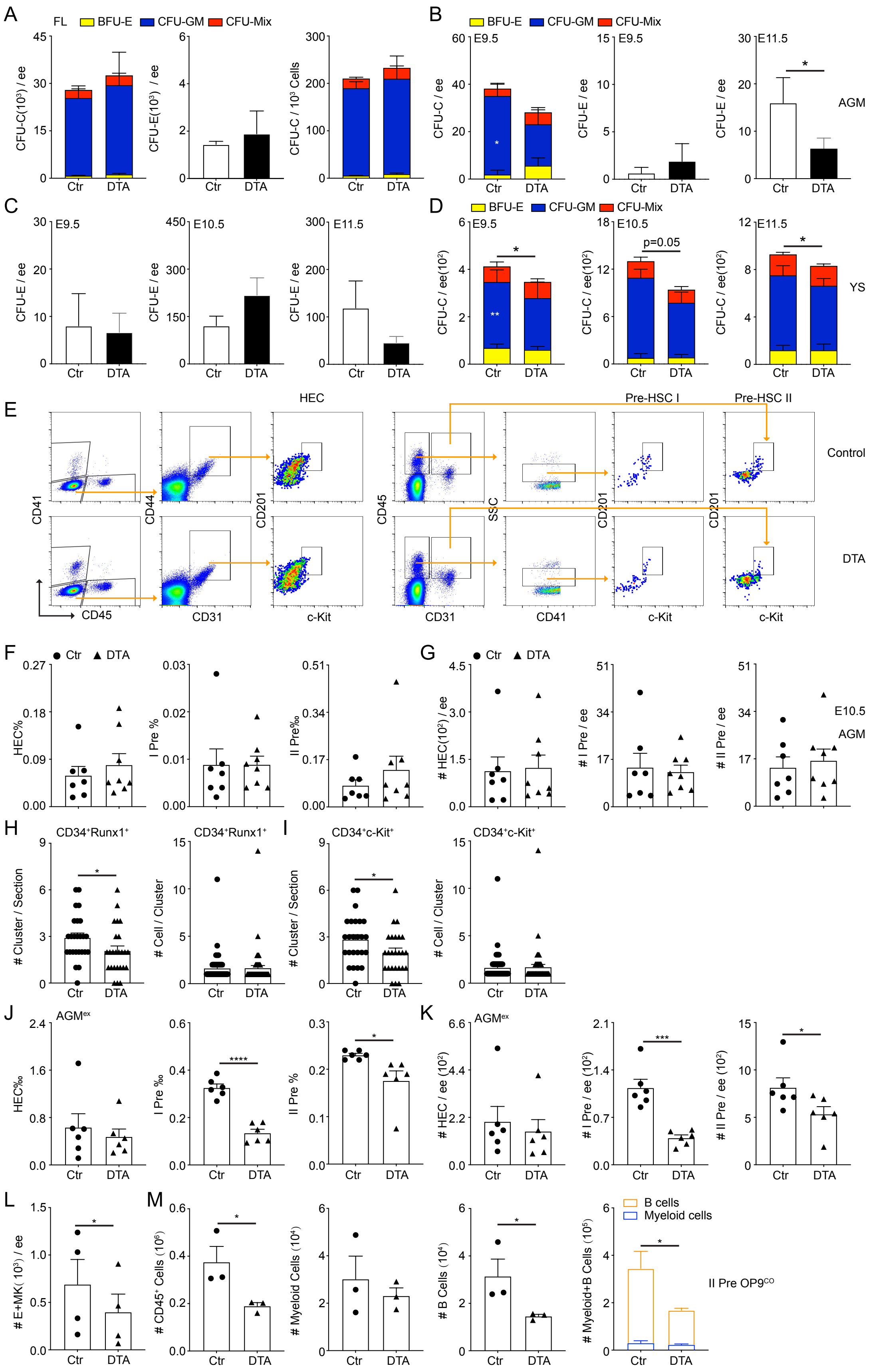

A
FL
BFU-E
CFU-GM
CFU-Mix
CFU-C(10³) / ee
CFU-E(10³) / ee
CFU-C / 10³ Cells
Ctr
DTA
B
E9.5
E11.5
CFU-C / ee
CFU-E / ee
AGM
C
E10.5
D
CFU-C / ee(10²)
p=0.05
YS
E
HEC
Pre-HSC I
Pre-HSC II
Control
DTA
CD41
CD45
CD44
CD31
CD201
c-Kit
SSC
CD41
F
HEC%
I Pre %
II Pre‰
G
HEC(10²) / ee
I Pre / ee
II Pre / ee
E10.5
AGM
H
CD34⁺Runx1⁺
Cluster / Section
Cell / Cluster
I
CD34⁺c-Kit⁺
J
AGMex
HEC‰
I Pre ‰
II Pre %
K
HEC / ee (10²)
I Pre / ee (10²)
II Pre / ee (10²)
L
E+MK(10³) / ee
M
CD45⁺ Cells (10⁶)
Myeloid Cells (10⁴)
B Cells (10⁴)
Myeloid+B Cells (10⁵)
B cells
Myeloid cells
II Pre OP9CO

Appendix Figure S3 (Related to Fig. 3 and 4) : The pre-Hematopoietic stem cells were unchanged in the E10.5 PF4-Cre;Rosa-DTA (DTA) AGM region. (A) Methylcellulose culture data showing the number of CFU-Cs, CFU-E per embryo equivalent (ee) and the number of CFU-Cs per 1000 cells in the E12.5 fetal liver. The number of each hematopoietic colony type is indicated by color bars. n=3 independent experiments. (B) The number of CFU-Cs and CFU-E in the E9.5 and E11.5 PF4-Cre;Rosa-DTA (DTA) AGM regions per embryo equivalent (ee) compared with control. n≥3 independent experiments, *p=0.0364. (C) Methylcellulose culture data showing the number of CFU-E per embryo equivalent (ee) in the E9.5-E11.5 yolk sac. n≥3 independent experiments. (D) The reduction number of CFU-Cs per embryo equivalent (ee) in the E9.5-E11.5 DTA yolk sac compared to control group. n≥3 independent experiments, *p<0.05, **p=0.0024. The number of each hematopoietic colony type is indicated by color bars. (E) The presentative gating strategy of flow analysis for hemogenic endothelial cells (HEC, $CD41^{-}CD45^{-}CD31^{+}CD44^{+}CD201^{+}c\text{-}Kit^{+}$) and pre-HSC I ($CD41^{low}CD45^{-}CD31^{+}CD201^{+}c\text{-}Kit^{+}$) and II ($CD45^{+}CD31^{+}CD201^{+}c\text{-}Kit^{+}$) in the E11.5 control and PF4-Cre;Rosa-DTA (DTA) AGM region. (F)The percentage of hemogenic endothelial cells(HEC) and pre-HSC I and II in the E10.5 control and PF4-Cre;Rosa-DTA (DTA) AGM region. n=7-8 fetuses from at least 3 litters/condition. (G) The HEC and pre-HSC I and II cell numbers failed to change in the E10.5 AGM region. n=7-8 fetuses from at least 3 litters/condition. (H) The hematopoietic clusters($CD34^{+}Runx1^{+}$) number per section(left) was reduced and the cell number of each hematopoietic cluster(right) was comparable in the E10.5 DTA AGM region compared with control group. n=25 sections of 3 fetuses from 3 litters. Statistical significance was determined by Mann-Whitney test. *p=0.0167. (I) The reduced hematopoietic clusters($CD34^{+}c\text{-}Kit^{+}$) number per section(left) and comparable cell number of each hematopoietic cluster(right) in the E10.5 DTA AGM region compared with control group. n=25 sections from 3 fetuses, Statistical significance was determined by Mann-Whitney test. *p=0.0231. (J-K) Flow cytometric analysis showing the reduced percentages and cell numbers of pre-HSC I and pre-HSC II in the DTA AGM^{ex} cells after 3 days explant culture. n=6 fetuses from at least 3 litters/condition, *p<0.05, ***p=0.0001, ****p<0.0001. Circles and triangles indicate individual recipients of control or DTA cells, respectively. (L) OP9 cocultures showing the reduction of erythroid

cell and Mk numbers from DTA pre-HSC II after 10 days coculture. n=4 independent experiments, *p=0.0329. E=Ter119^{+} cells, Mk=CD41^{+}CD45^{-}Ter119^{-} cells. (M) Pre-HSC II derived hematopoietic cells after 10 days OP9 cocultures. n=3 independent experiments, *p<0.05. Myeloid cells=CD45^{+}CD11b^{+}/Gr1^{+}Ter119^{-}CD41^{-} cells, B cells=CD45^{+}CD-19^{+}CD11b^{-}/Gr1^{-}Ter119^{-}CD41^{-} cells.

Data Information: For all analysis above bars represent mean±SEM. Statistical significance was determined by unpaired Student's t-test unless the statistical test was indicated.

A

Stage	# embryos	Tissues	Exp(n)	Fractions	#Collecting Cell	#Analyzed Cell
E11.5(42-45 sp)	10	AGM	1	$CD45^-CD41^+CD42^+$	48	44
				$CD45^-CD41^+CD42^+CD226^+$	48	46
				$CD45^-CD41^+CD42^+CD226^-$	48	48
E11.5(41-44 sp)	9	AGM	1	$CD45^-CD41^+CD42^+$	96	71
E9.5(19-21 sp)	4	YS	1	$CD41^{low}cKit^+$	48	48
					96	94
Total	23	---	3	---	384	351

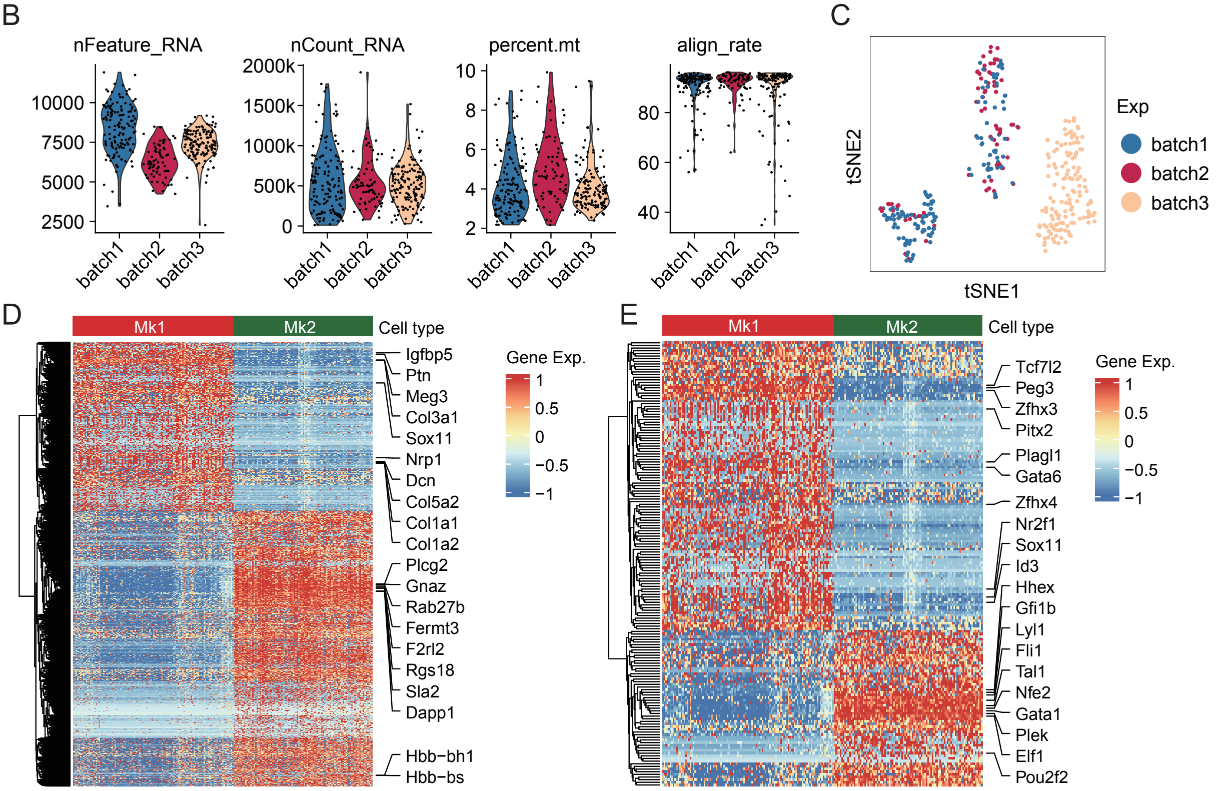

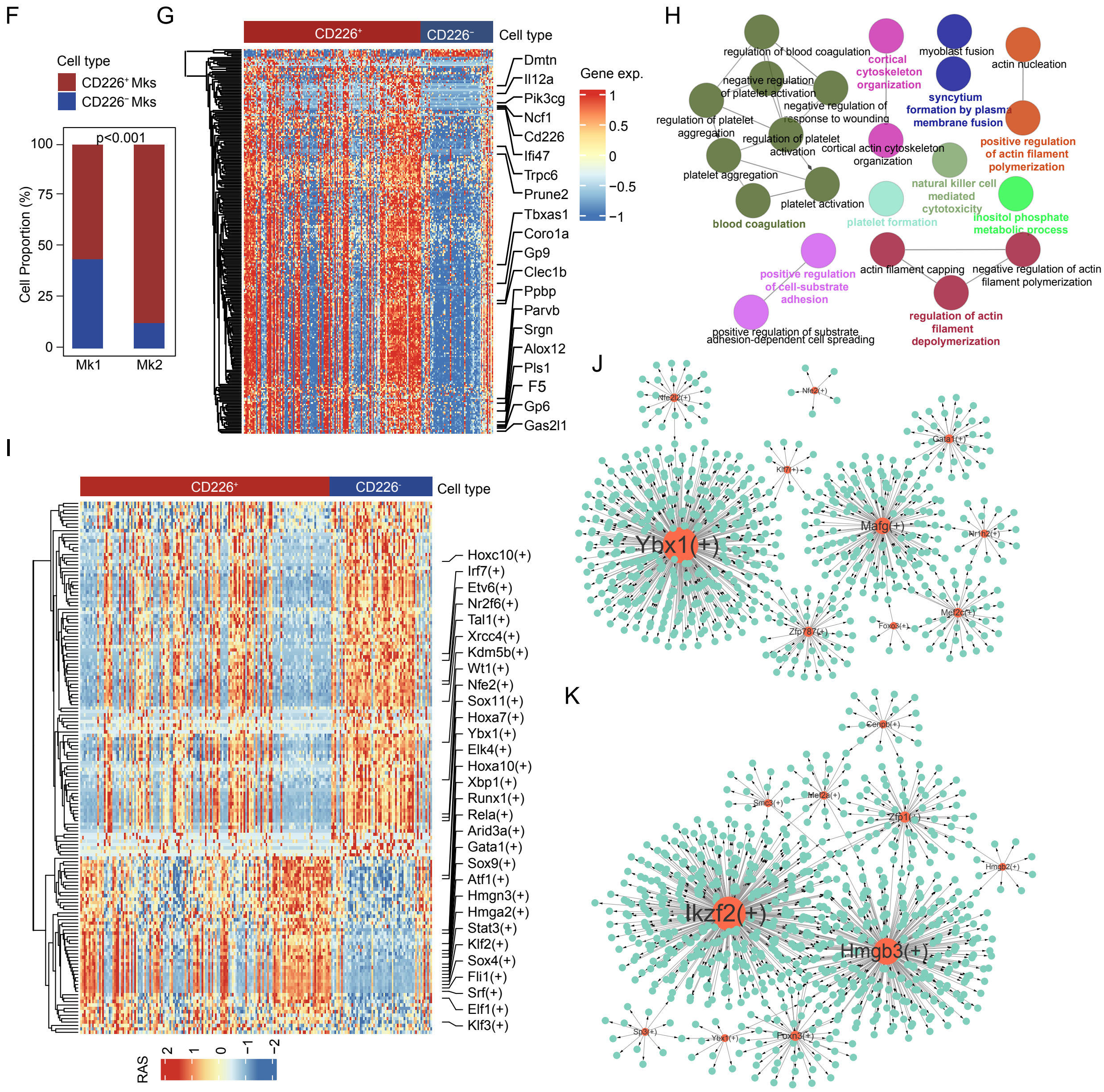
F
G
H
I
J
K
Cell type
CD226⁺ Mks
CD226⁻ Mks
p<0.001
Cell Proportion (%)
Mk1
Mk2
CD226⁺
CD226⁻
Gene exp.
Dmtn
Il12a
Pik3cg
Ncf1
Cd226
Ifi47
Trpc6
Prune2
Tbxas1
Coro1a
Gp9
Clec1b
Ppbp
Parvb
Srgn
Alox12
Pls1
F5
Gp6
Gas2l1
regulation of blood coagulation
negative regulation of platelet activation
negative regulation of response to wounding
regulation of platelet aggregation
regulation of platelet activation
platelet aggregation
platelet activation
blood coagulation
cortical cytoskeleton organization
cortical actin cytoskeleton organization
myoblast fusion
syncytium formation by plasma membrane fusion
actin nucleation
positive regulation of actin filament polymerization
natural killer cell mediated cytotoxicity
platelet formation
inositol phosphate metabolic process
positive regulation of cell-substrate adhesion
positive regulation of substrate adhesion-dependent cell spreading
actin filament capping
negative regulation of actin filament polymerization
regulation of actin filament depolymerization
Hoxc10(+)
Irf7(+)
Etv6(+)
Nr2f6(+)
Tal1(+)
Xrcc4(+)
Kdm5b(+)
Wt1(+)
Nfe2(+)
Sox11(+)
Hoxa7(+)
Ybx1(+)
Elk4(+)
Hoxa10(+)
Xbp1(+)
Runx1(+)
Rela(+)
Arid3a(+)
Gata1(+)
Sox9(+)
Atf1(+)
Hmgn3(+)
Hmga2(+)
Stat3(+)
Klf2(+)
Sox4(+)
Fli1(+)
Srf(+)
Elf1(+)
Klf3(+)
RAS
Ybx1(+)
Mafg(+)
Ikzf2(+)
Hmgb3(+)

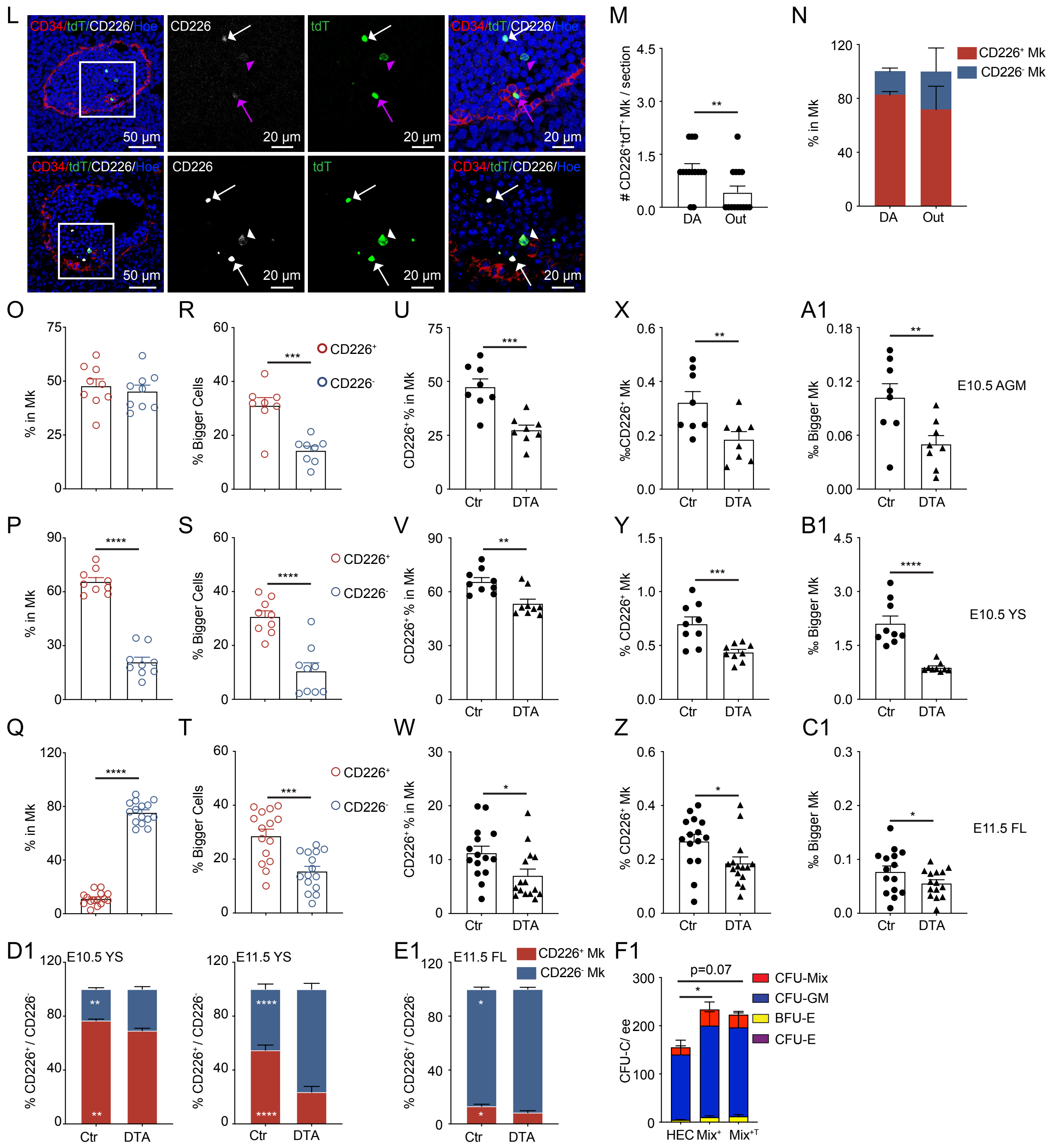

Appendix Figure S4 (Related to Fig.5 and 6) : Single-cell RNA-sequencing analysis identifying the differences between distinct subfractions of megakaryocytes and flow analysis confirmed the alteration of CD226⁺ megakaryocytes in the DTA AGM regions. (A) The information of samples from E11.5 AGM regions and E9.5 yolk sac. (B) Quality control by nFeature, nCount, and percentage of mitochondrial and align rate of

the library. mt.=mitochondria. (C) TSNE graphs showing no batch differences. (D-E) The differences in gene expression and transcription factor between Mk1 and Mk2. (F) The overlap of CD226$^+$ cluster cells and CD226$^-$ cluster cells with Mk1 and Mk2. P<0.001 was calculated by Chi-Squared test. (G) The different expression genes(DEG) between CD226$^+$ cluster cells and CD226$^-$ cluster cells. (H) The function enrichment analysis based on the DEG. (I) The distinct activity of transcription factors(regulons) between CD226$^+$ cluster cells and CD226$^-$ cluster cells. RAS=regulon activity score. (J-K) The regulatory networks of transcription factor target genes with top 10 regulon activity scores(RAS) in CD226$^+$ cluster cells and CD226$^-$ cluster of cells. (L) Immunostaining of cryosections for CD226 in Mks in the E10.5 embryos. Scale bar presents 50 μm or 20 μm. Red=CD34, Green=tdTomato(tdT), White=CD226, Blue=Hoechst. White arrowheads indicate CD226$^+$tdT$^+$ Mk cells and purple arrowheads indicate CD226$^-$tdT$^+$ Mk cells. White arrows indicate CD226$^+$tdT$^+$ platelets and purple arrows indicate CD226$^-$ tdT$^+$ platelets. (M) The number of CD226$^+$tdT$^+$ Mk cells in and out (0-150 μm) of aorta. n=14 sections of 3 fetuses from 3 litters. Statistical significance was determined by Mann-Whitney test. **p=0.0060. (N) The percentages of CD226$^+$tdT$^+$ and CD226$^-$tdT$^+$ Mk cells in and out (0-150 μm) of aorta. n=3 independent experiments. (O-Q) The percentages of CD226$^+$ and CD226$^-$ cells in the Mks in E10.5 AGM region(O), yolk sac (P) and E11.5 fetal liver(Q). n=9-15 independent experiments, ****p<0.0001. (R-T) The percentages of bigger cell sizes(SSH^{high}) in the CD226$^+$ and CD226$^-$ fraction of Mks in E10.5 AGM region(R), yolk sac(S) and E11.5 fetal liver(T). n=8-15 independent experiments, ***p<0.001, ****p<0.0001. (U-W) Percentages of CD226$^+$ in Mks in the E10.5 control and DTA AGM region(U), yolk sac(V) and E11.5 fetal liver(W). n=8-15 fetuses from at least 3 litters/condition, *p=0.0109, **p=0.0011, ***p=0.0002. (X-Z) The decreased percentage of CD226$^+$ Mks in the E10.5 AGM region(X), yolk sac(Y) and E11.5 fetal liver(Z) after PF4 deletion compared to corresponding control. n=8-15 fetuses from at least 3 litters/condition, *p=0.0126, **p=0.008, ***p=0.0008. (A1-C1) The percentage of bigger Mks in the E10.5 AGM region(A1), yolk sac(B1) and E11.5 fetal liver (C1). n=8-15 fetuses from at least 3 litters/condition, *p=0.0473, **p=0.0062, ****p<0.0001. (D1-E1) The ratios between CD226$^+$ and CD226$^-$ percentage in the E10.5-E11.5 yolk sac (D1) and E11.5 fetal liver (E1). n=8-15 fetuses from at least 3 litters/condition, *p<0.05, **p<0.01,

****$p<0.0001$. Yolk sac=YS, FL=fetal liver. (F1) CFU-Cs from $CD45^+$ cells in the OP9-DL1 coculture system showing the enhancement of Mks with or without transwells. HEC= Hemogenic Endothelial cells ($CD41^-CD45^-CD31^+CD44^+$), Mix^+=HEC+$CD226^+$Mks, Mix^{+T}=HEC+ $CD226^+$Mks+Transwells. n=4 independent experiments. Statistical significance was determined by paired Student's t-test. *$p<0.05$. The number of each hematopoietic colony type is indicated by color bars.

Data Information: For all analysis above bars represent mean±SEM. Statistical significance was determined by unpaired Student's t-test unless the statistical test was indicated.

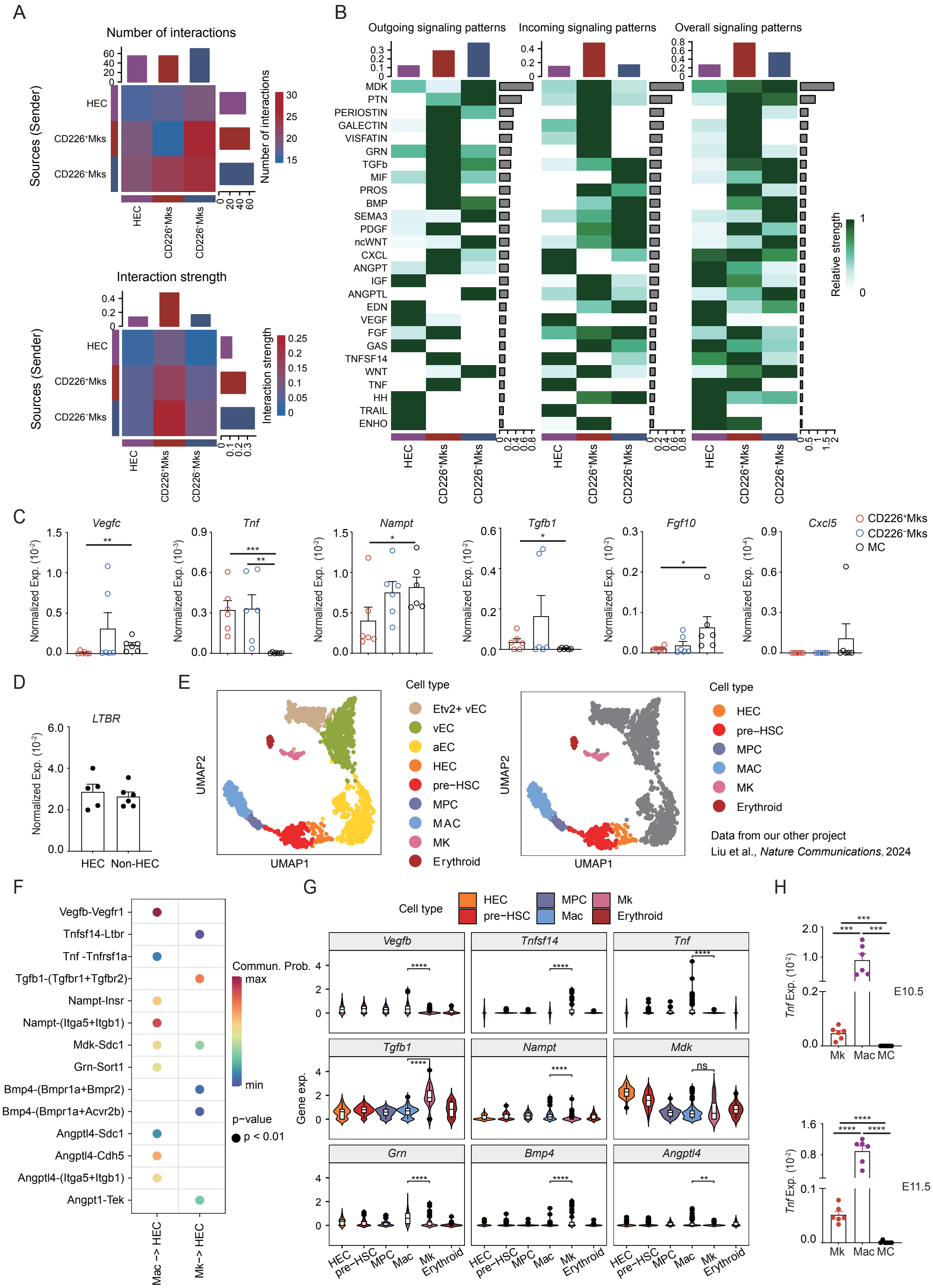

A
Number of interactions
Sources (Sender)
HEC
CD226⁺Mks
CD226⁻Mks
Number of interactions
Interaction strength
B
Outgoing signaling patterns
Incoming signaling patterns
Overall signaling patterns
MDK
PTN
PERIOSTIN
GALECTIN
VISFATIN
GRN
TGFb
MIF
PROS
BMP
SEMA3
PDGF
ncWNT
CXCL
ANGPT
IGF
ANGPTL
EDN
VEGF
FGF
GAS
TNFSF14
WNT
TNF
HH
TRAIL
ENHO
Relative strength
C
Vegfc
Tnf
Nampt
Tgfb1
Fgf10
Cxcl5
Normalized Exp.
CD226⁺Mks
CD226⁻Mks
MC
D
LTBR
Normalized Exp.
HEC
Non-HEC
E
Cell type
Etv2+ vEC
vEC
aEC
HEC
pre–HSC
MPC
MAC
MK
Erythroid
UMAP1
UMAP2
Data from our other project
Liu et al., Nature Communications, 2024
F
Vegfb-Vegfr1
Tnfsf14-Ltbr
Tnf -Tnfrsf1a
Tgfb1-(Tgfbr1+Tgfbr2)
Nampt-Insr
Nampt-(Itga5+Itgb1)
Mdk-Sdc1
Grn-Sort1
Bmp4-(Bmpr1a+Bmpr2)
Bmp4-(Bmpr1a+Acvr2b)
Angptl4-Sdc1
Angptl4-Cdh5
Angptl4-(Itga5+Itgb1)
Angpt1-Tek
Commun. Prob.
max
min
p−value
p < 0.01
Mac -> HEC
Mk-> HEC
G
Cell type
HEC
pre−HSC
MPC
Mac
Mk
Erythroid
Vegfb
Tnfsf14
Tnf
Tgfb1
Nampt
Mdk
Grn
Bmp4
Angptl4
Gene exp.
H
Tnf Exp.
Mk
Mac
MC
E10.5
E11.5

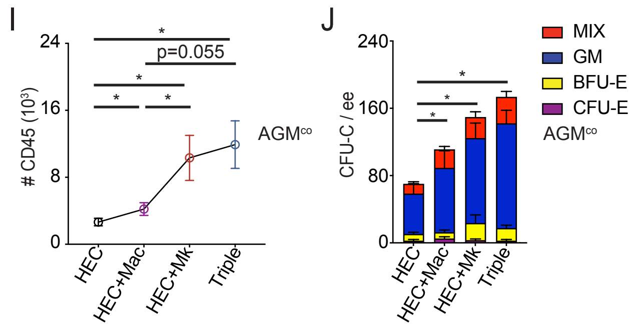

Appendix Figure S5: Related to Fig.7 Single-cell RNA-sequencing analysis supports the synergy of megakaryocytes and macrophages on the hematopoiesis in the embryos. (A) The interaction number and strength between Mks and HECs. (B) The signaling pathways were relative to the interaction between Mks and HECs. (C) qRT-PCR confirmed the expression of selected genes in the $CD226^+$ Mks and $CD226^-$ Mks as well as mesenchymal cells(MC). n=3 independent experiments, *p<0.05, **p<0.01, ***p=0.0005. (D) The expression of LTBR in the $CD44^+$ EC(HEC, $CD41^-CD45^-CD31^+CD44^+$), and $CD44^-$ EC (non-HEC) by qRT-PCR. n=3 independent experiments. (E) The UMAP data from our other project. Left one indicated all hematopoietic-related cells. Colored clusters on the right were selected to use in this study. (F) Comparative analysis showing the specific pathways of interaction between Mac/Mk and HECs. Statistical significance was determined by one-sided permutation test. (G) Gene expression showing Tnfsf14 and Tnf specifically expressed more highly in the Mks and Macs, respectively. The box plot's central band marks the median, boxes mark the first and third quartiles. n=3 independent experiments. Statistical significance was determined by Wilcoxon test. **p<0.01, ****p<0.0001. (H) qRT-PCR confirmed the higher expression of Tnf in the Macs compared with Mks/MCs in the E10.5-E11.5 AGM region. n=3 independent experiments, ***p<0.001, ****p<0.0001. (I) $CD45^+$ hematopoietic cells derived from E10.5 AGM hemogenic endothelial cells with Macrophage and/or Megakaryocytes by OP9-DL1 coculture system for 3 days. n=4 independent experiments. Statistical significance was determined by paired Student's t-test. *p<0.05. Mac= Macrophage, Mk=Megakaryocyte. Triple=HEC+Mac+Mk. (J) CFU-Cs from E10.5 AGM hemogenic endothelial cells with Macrophage and/or Megakaryocytes by OP9-DL1 coculture system for 3 days. n=4 independent experiments. Statistical significance was

determined by paired Student's t-test. *$p<0.05$. Mac= Macrophage, Mk=Megakaryocyte. Triple=HEC+Mac+Mk. Colors indicates type of CFU-Cs. The number of each hematopoietic colony type is indicated by color bars.

Data Information: For all analysis above bars represent mean±SEM. Statistical significance was determined by unpaired Student's t-test unless the statistical test was indicated.

Appendix Table S1: Primers for genotype and qRT-PCR.

Oligonucleotides	SOURCE
DTA-Forward: GTTATCAGTAAGGGAGCTGCAGTGG	Tsingke
DTA Control-Reverse: GGCGGATCACAAGCAATAATAACC	Tsingke
DTA MT-Reverse: AAGACCGCGAAGAGTTTGTCCTC	Tsingke
Pf4 Forward: CCAAGTCCTACTGTTTCTCACTC	Tsingke
Pf4 Reverse: TGCACAGTCAGCAGGTT	Tsingke
Pf4 Control-Forward: CTAGGCCACAGAATTGAAAGATCT	Tsingke
Pf4 Control-Reverse: GTAGGTGGAAATTCTAGCATCATCC	Tsingke
tdT Control -Forward: AAGGGAGCTGCAGTGGAGTA	Tsingke
tdT Control -Reverse: CCGAAAATCTGTGGGAAGTC	Tsingke
tdT MT-Reverse: GGCATTAAAGCAGCGTATCC	Tsingke
tdT MT-Forward: CTGTTCCTGTACGGCATGG	Tsingke
Tnfsf14 Forward: GTTTCTCCTGAGACTGCATCAA	Tsingke
Tnfsf14 Reverse: TGGCTCCTGTAAGATGTGCTG	Tsingke
Lgals9 Forward: ATGCCCTTTGAGCTTTGCTTC	Tsingke
Lgals9 Reverse: AACTGGACTGGCTGAGAGAAC	Tsingke
Ltbr Forward: TGGTGCCCCCTTATCGCATA	Tsingke
Ltbr Reverse: TGCATACCGCAAAGACAAACTC	Tsingke
Angpt1 Forward: CACATAGGGTGCAGCAACCA	Tsingke
Angpt1 Reverse: CGTCGTGTTCTGGAAGAATGA	Tsingke
Vegfc Forward: GAGGTCAAGGCTTTTGAAGGC	Tsingke
Vegfc Reverse: CTGTCCTGGTATTGAGGGTGG	Tsingke
Tnf Forward: CCCTCACACTCAGATCATCTTCT	Tsingke
Tnf Reverse: GCTACGACGTGGGCTACAG	Tsingke
Nampt Forward: GCAGAAGCCGAGTTCAACATC	Tsingke
Nampt Reverse: TTTTCACGGCATTCAAAGTAGGA	Tsingke
Tgfb1 Forward: CTCCCGTGGCTTCTAGTGC	Tsingke
Tgfb1 Reverse: GCCTTAGTTTGGACAGGATCTG	Tsingke
Fgf10 Forward: TTTGGTGTCTTCGTTCCCTGT	Tsingke
Fgf10 Reverse: TAGCTCCGCACATGCCTTC	Tsingke
Cxcl5 Forward: GTTCCATCTCGCCATTCATGC	Tsingke
Cxcl5 Reverse: GCGGCTATGACTGAGGAAGG	Tsingke
