# Supplementary figures and images for: A subset of megakaryocytes regulates development of hematopoietic stem cell precursors
Source: EMBO J. 2024 Apr 5;43(9):1722–39. doi: 10.1038/s44318-024-00079-4 (PMC11065989; doi:10.1038/s44318-024-00079-4)

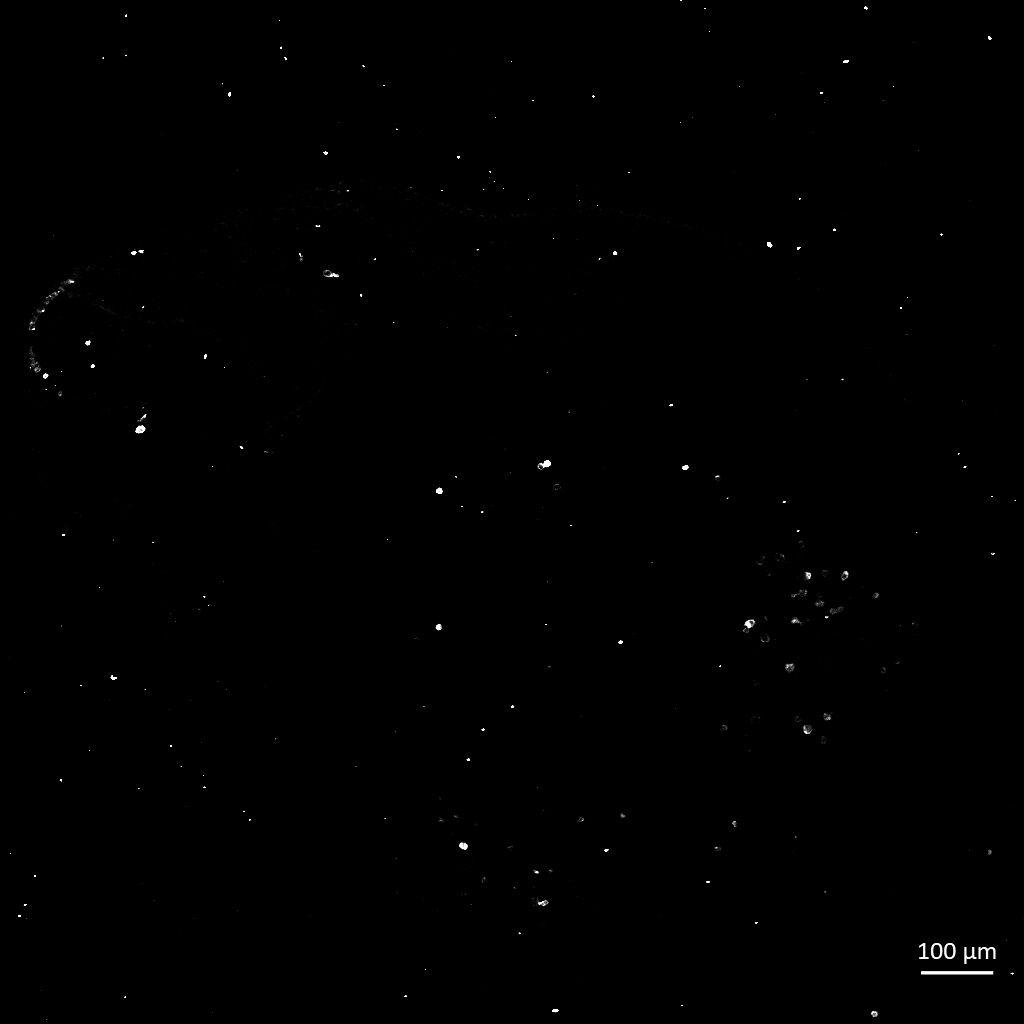

Supplement: Supplementary file 6 — Source data Fig. 1 [file 44318_2024_79_MOESM6_ESM.zip › source data-Figure 1/source data-Figure 1I/Figure I-1/Figure I-1_tdT.jpg]

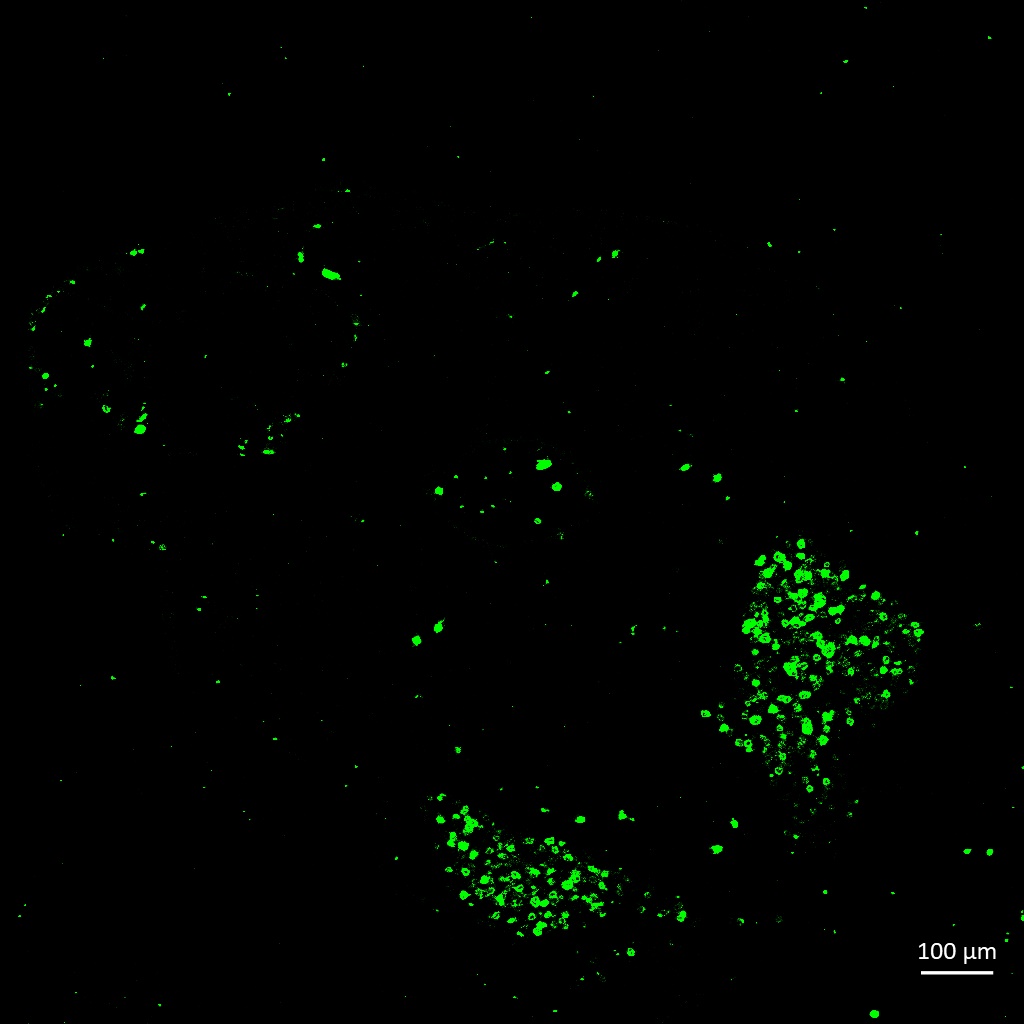

Supplement: Supplementary file 6 — Source data Fig. 1 [file 44318_2024_79_MOESM6_ESM.zip › source data-Figure 1/source data-Figure 1I/Figure I-1/Figure I-1_CD41.jpg]

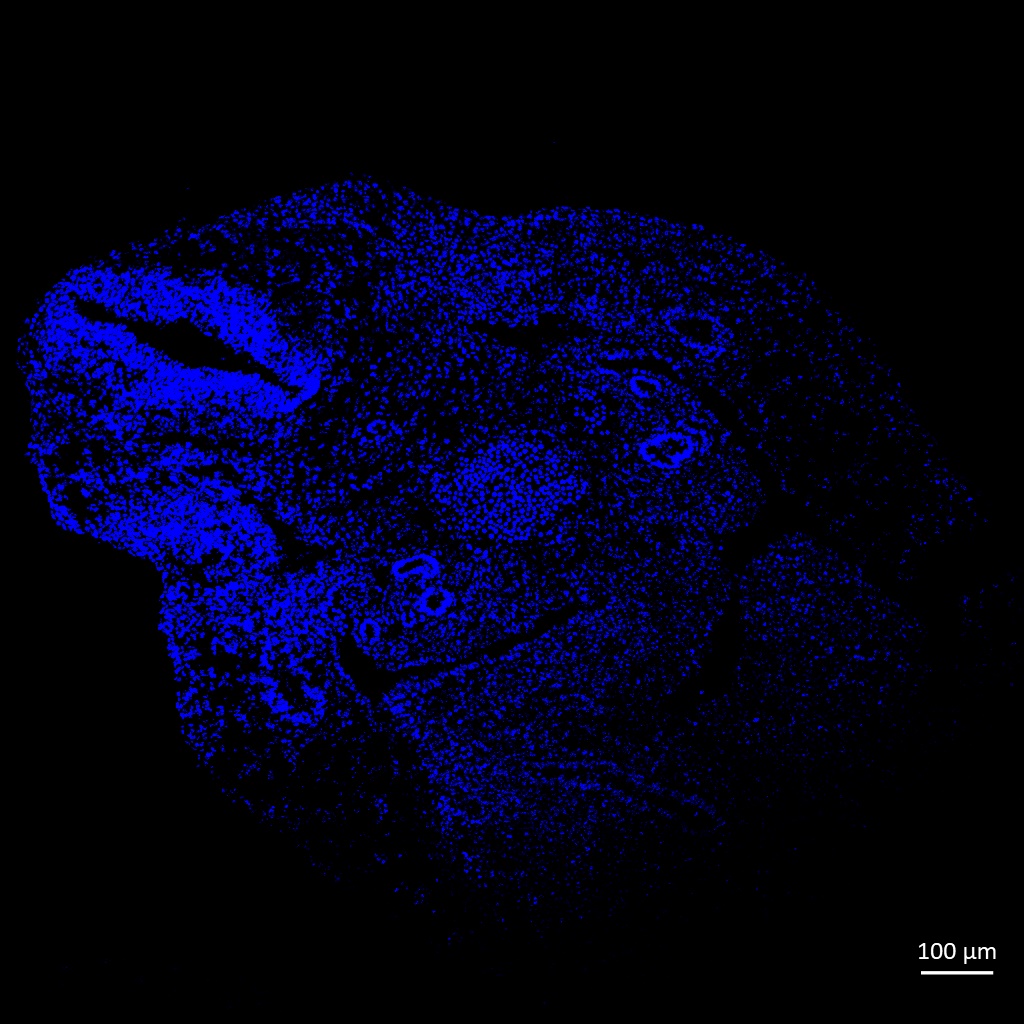

Supplement: Supplementary file 6 — Source data Fig. 1 [file 44318_2024_79_MOESM6_ESM.zip › source data-Figure 1/source data-Figure 1I/Figure I-1/Figure I-1_Hoechst.jpg]

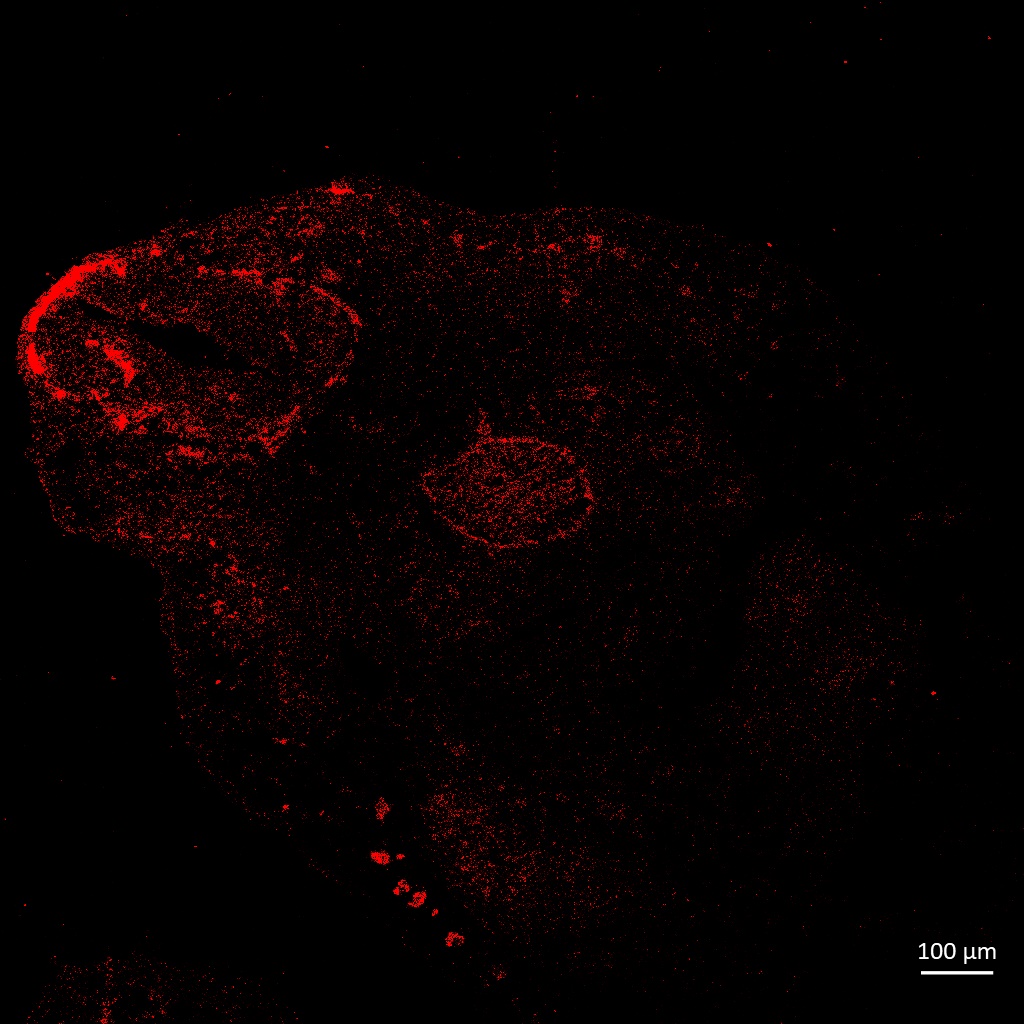

Supplement: Supplementary file 6 — Source data Fig. 1 [file 44318_2024_79_MOESM6_ESM.zip › source data-Figure 1/source data-Figure 1I/Figure I-1/Figure I-1_CD34.jpg]

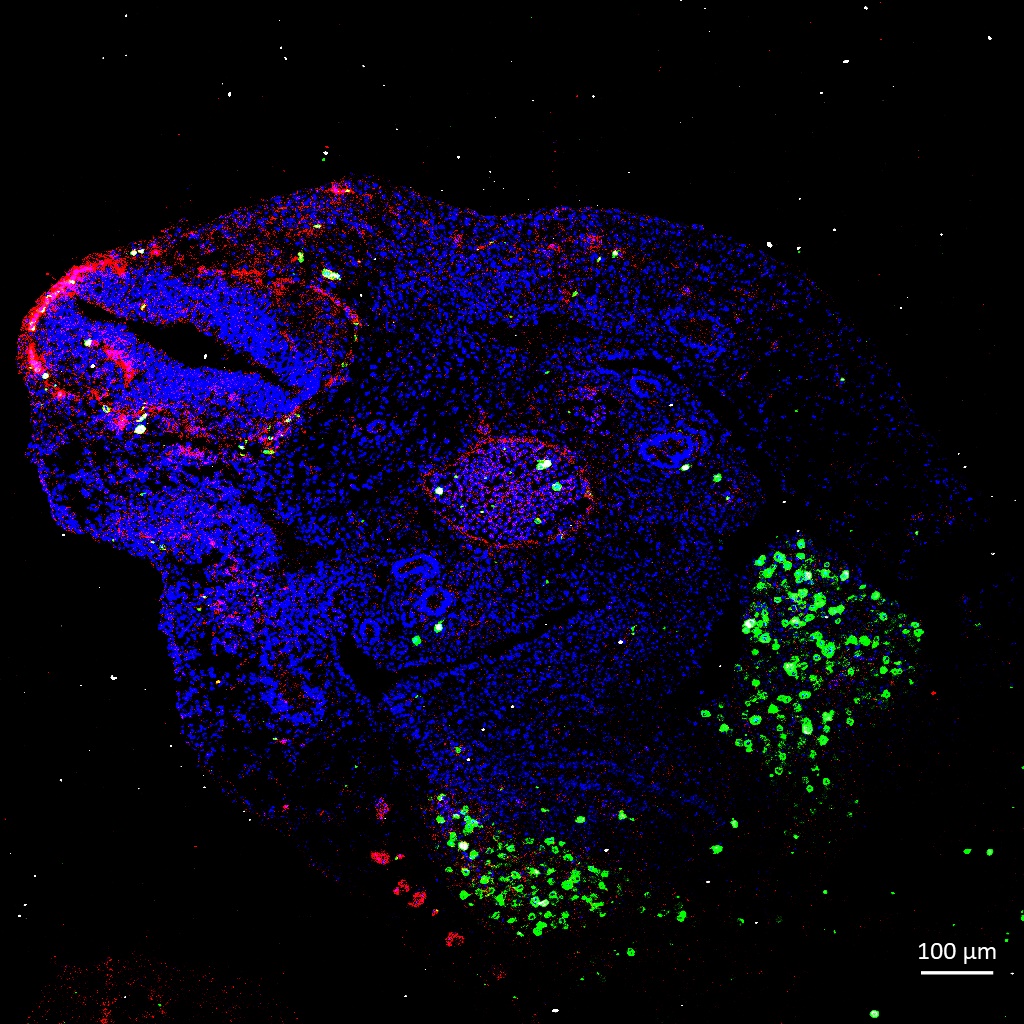

Supplement: Supplementary file 6 — Source data Fig. 1 [file 44318_2024_79_MOESM6_ESM.zip › source data-Figure 1/source data-Figure 1I/Figure I-1/Figure I-1_Merge.jpg]

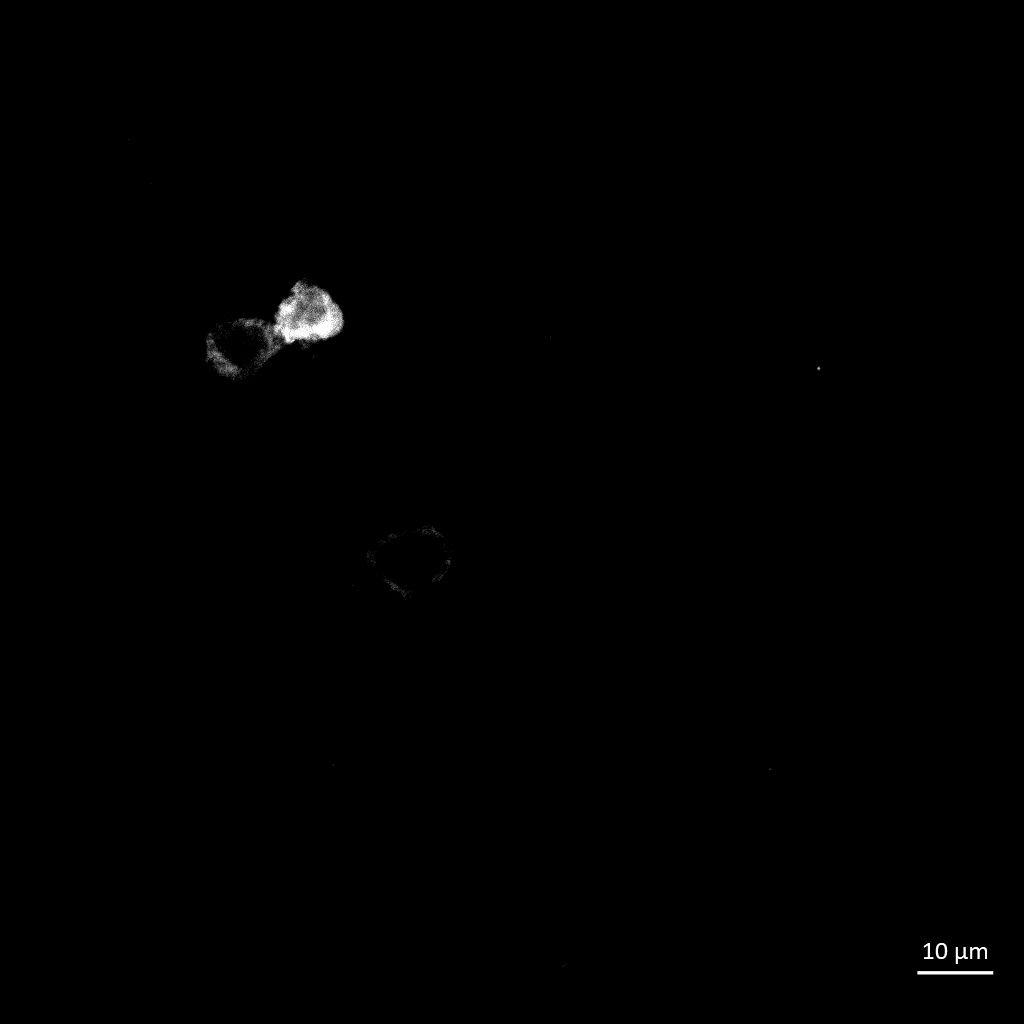

Supplement: Supplementary file 6 — Source data Fig. 1 [file 44318_2024_79_MOESM6_ESM.zip › source data-Figure 1/source data-Figure 1I/Figure I-2/Figure I-2_tdT.jpg]

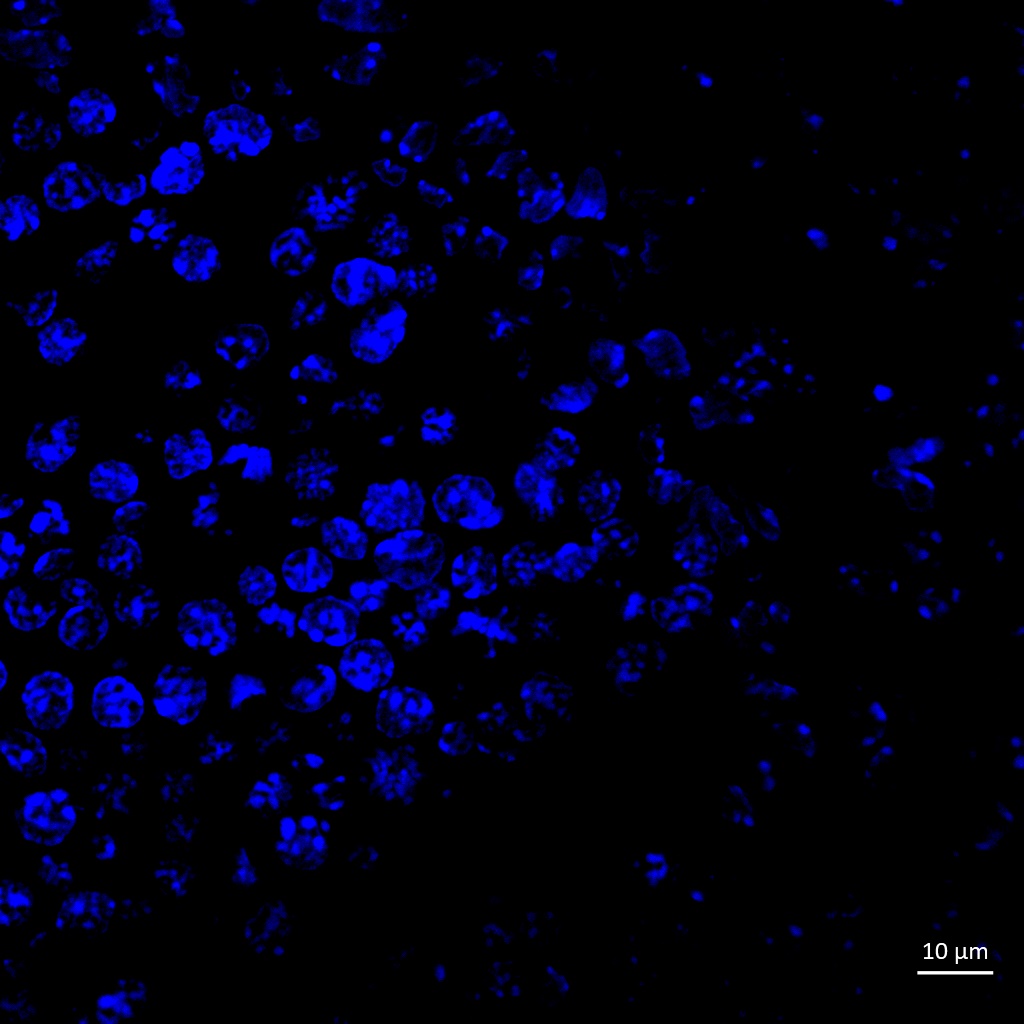

Supplement: Supplementary file 6 — Source data Fig. 1 [file 44318_2024_79_MOESM6_ESM.zip › source data-Figure 1/source data-Figure 1I/Figure I-2/Figure I-2_Hoechst.jpg]

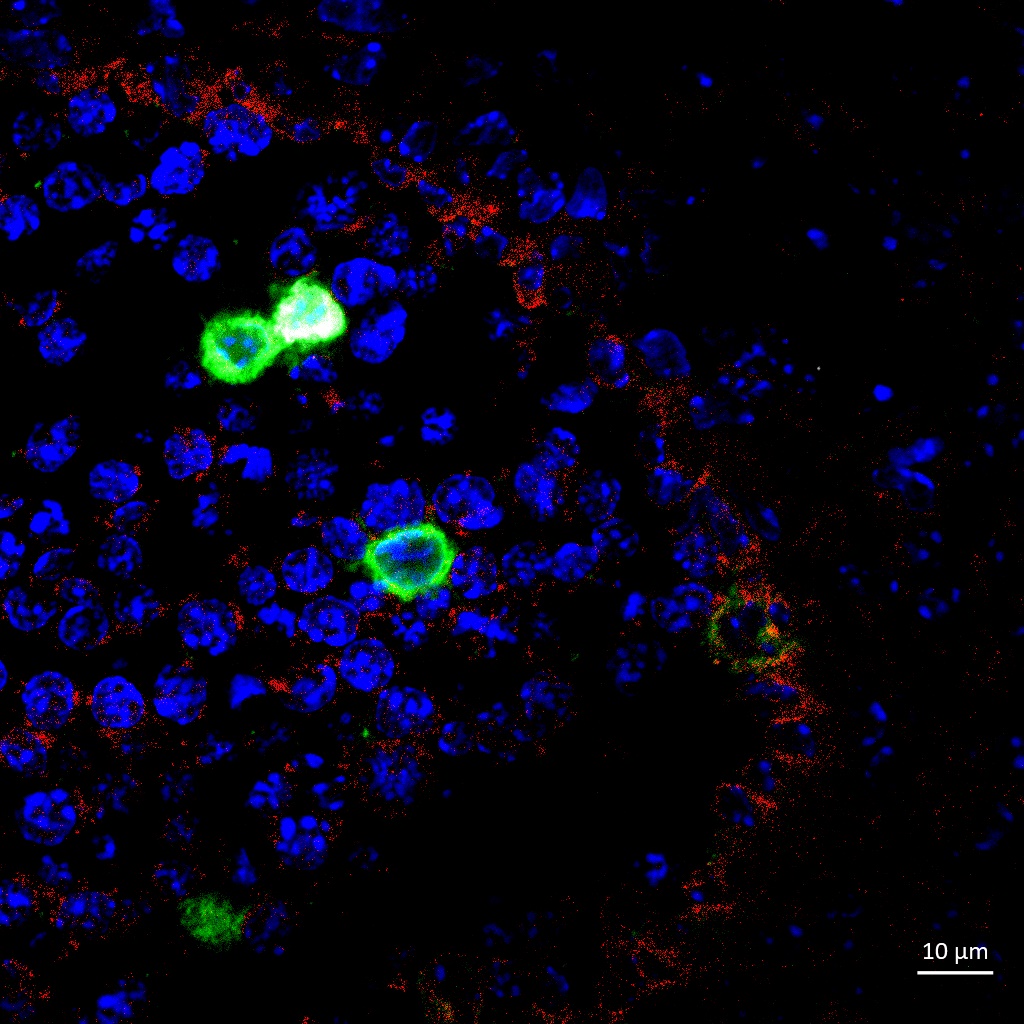

Supplement: Supplementary file 6 — Source data Fig. 1 [file 44318_2024_79_MOESM6_ESM.zip › source data-Figure 1/source data-Figure 1I/Figure I-2/Figure I-2_Merge.jpg]

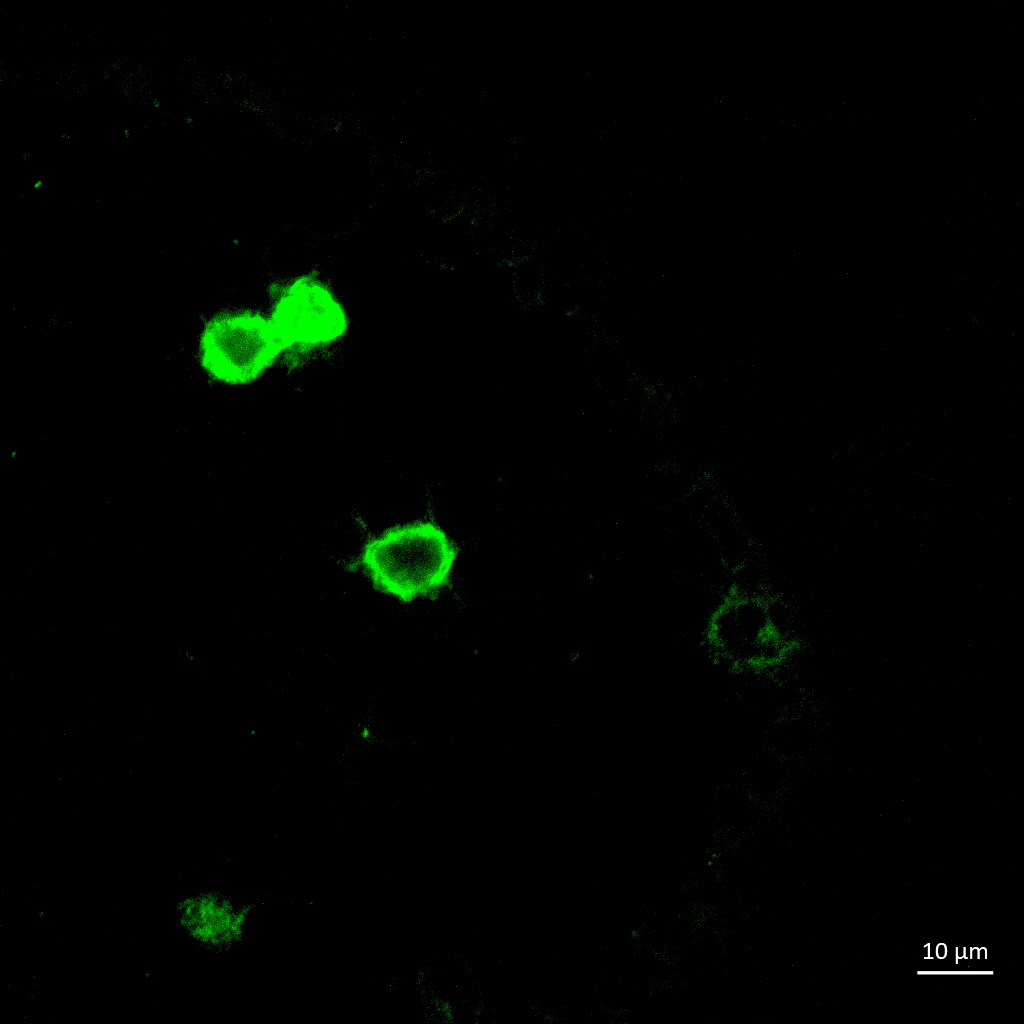

Supplement: Supplementary file 6 — Source data Fig. 1 [file 44318_2024_79_MOESM6_ESM.zip › source data-Figure 1/source data-Figure 1I/Figure I-2/Figure I-2_CD41.jpg]

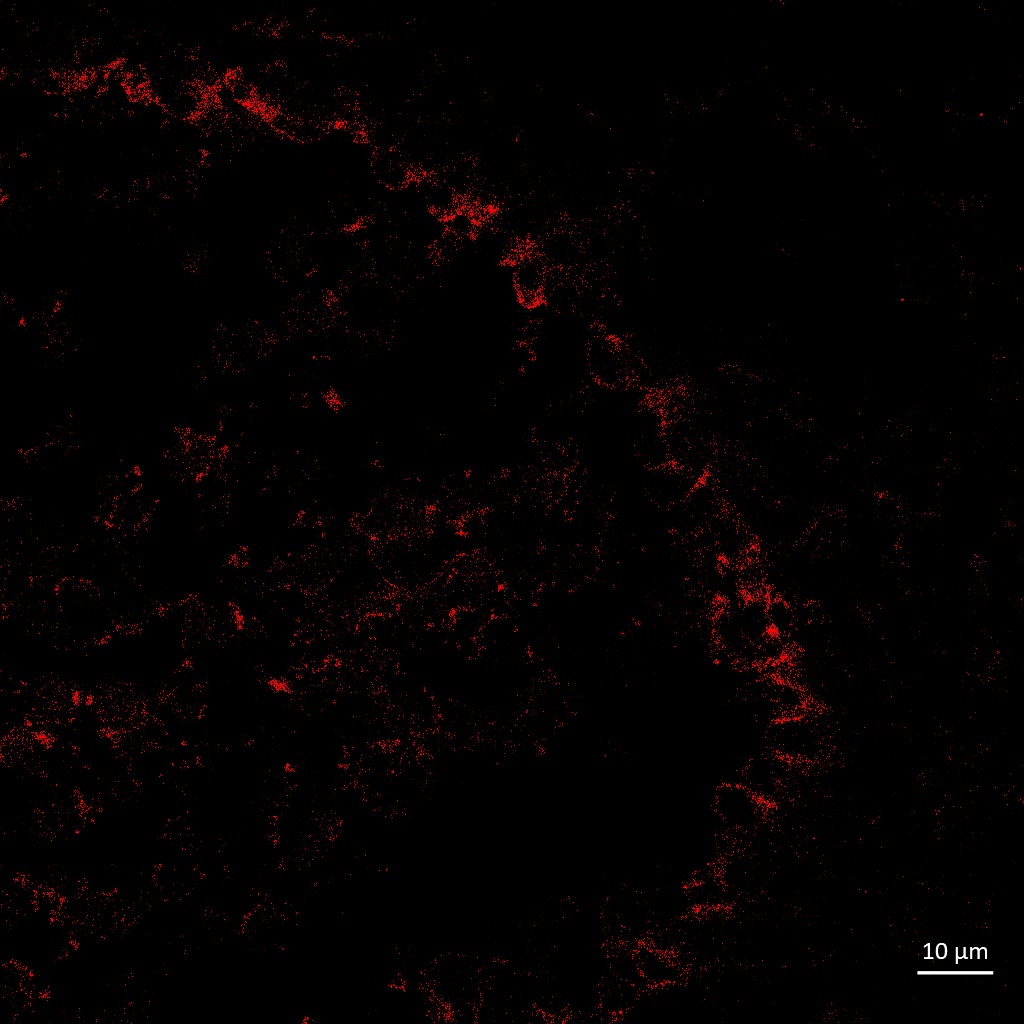

Supplement: Supplementary file 6 — Source data Fig. 1 [file 44318_2024_79_MOESM6_ESM.zip › source data-Figure 1/source data-Figure 1I/Figure I-2/Figure I-2_CD34.jpg]

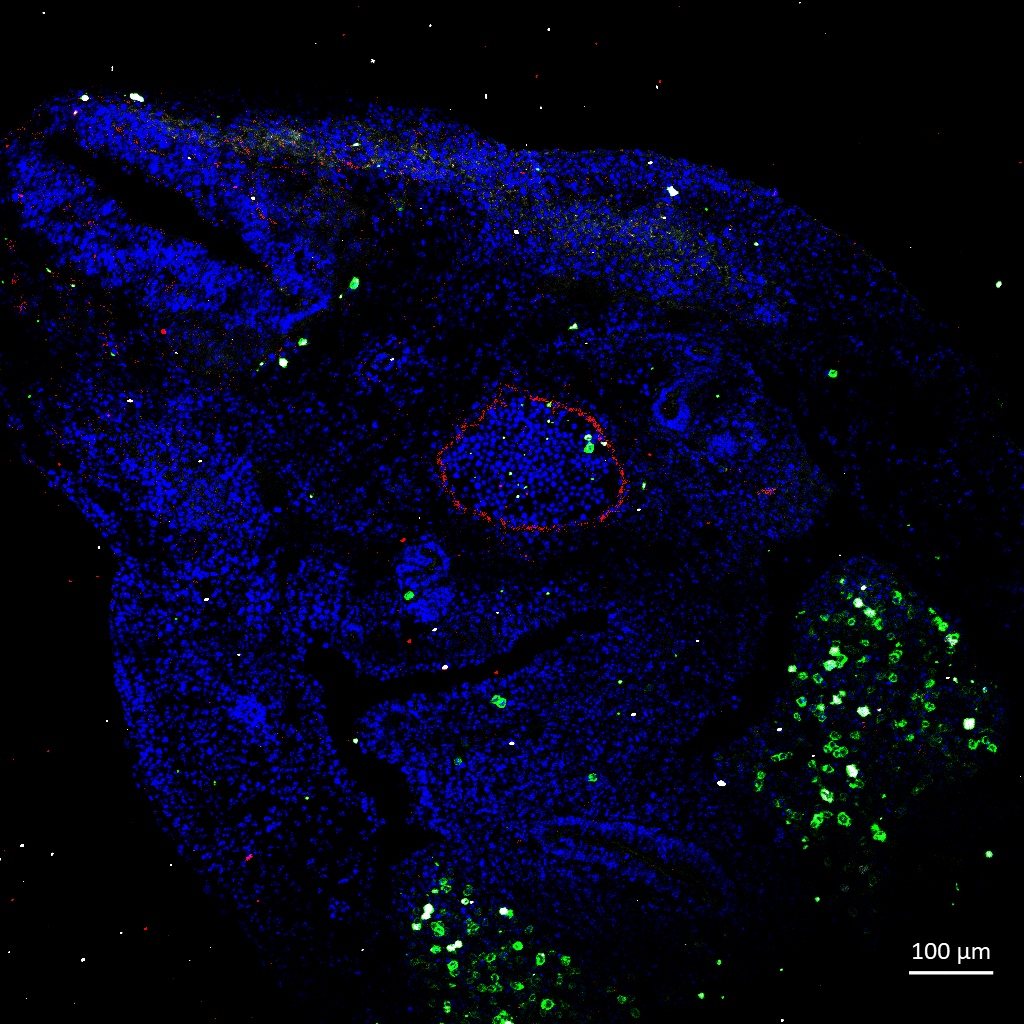

Supplement: Supplementary file 6 — Source data Fig. 1 [file 44318_2024_79_MOESM6_ESM.zip › source data-Figure 1/source data-Figure 1I/Figure I-3/Figure I-3_Merge.jpg]

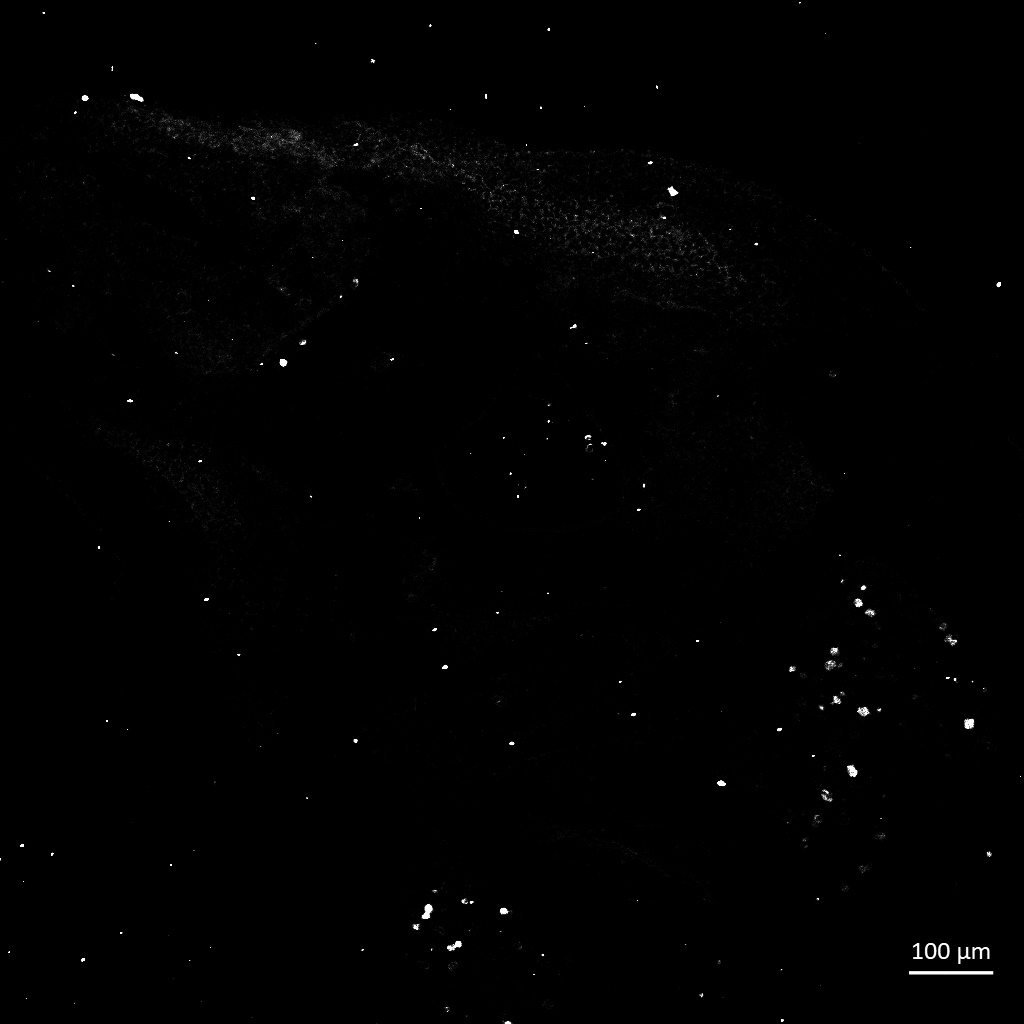

Supplement: Supplementary file 6 — Source data Fig. 1 [file 44318_2024_79_MOESM6_ESM.zip › source data-Figure 1/source data-Figure 1I/Figure I-3/Figure I-3_tdT.jpg]

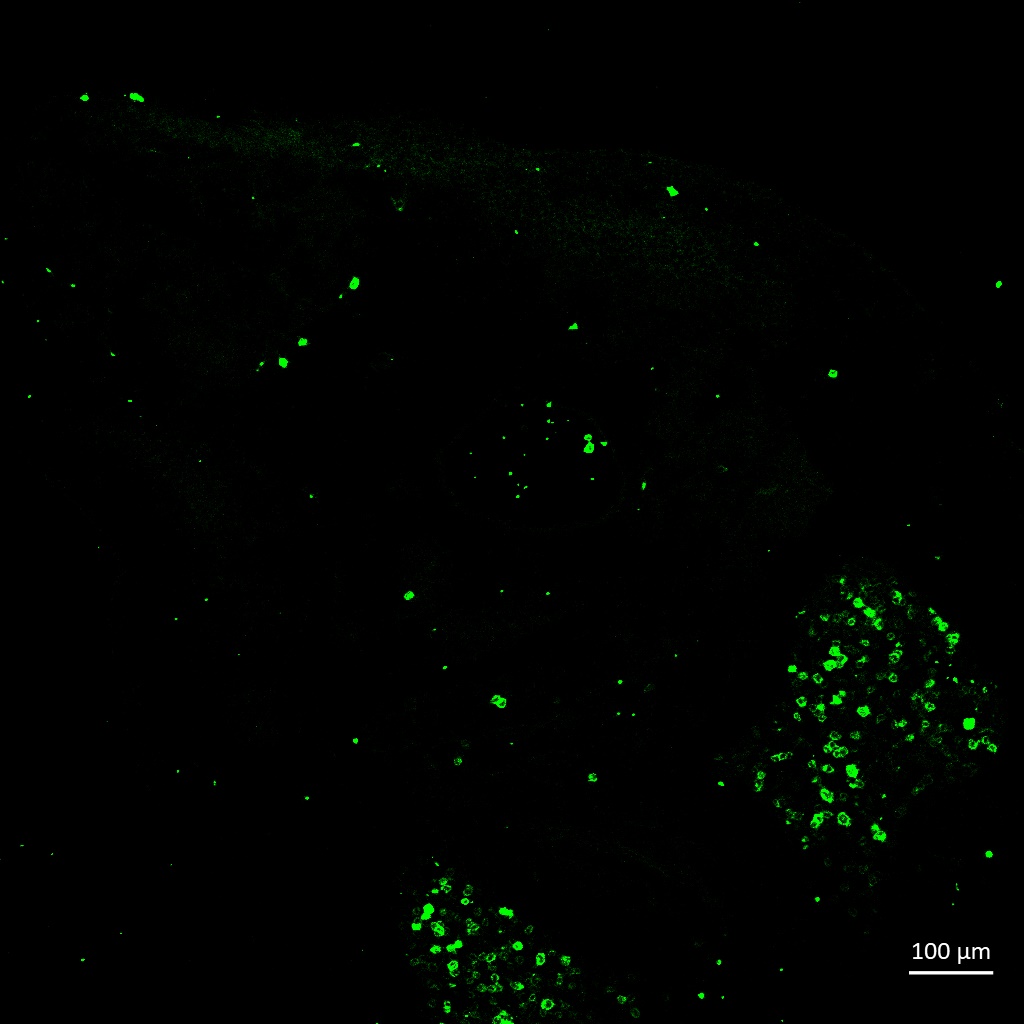

Supplement: Supplementary file 6 — Source data Fig. 1 [file 44318_2024_79_MOESM6_ESM.zip › source data-Figure 1/source data-Figure 1I/Figure I-3/Figure I-3_CD41.jpg]

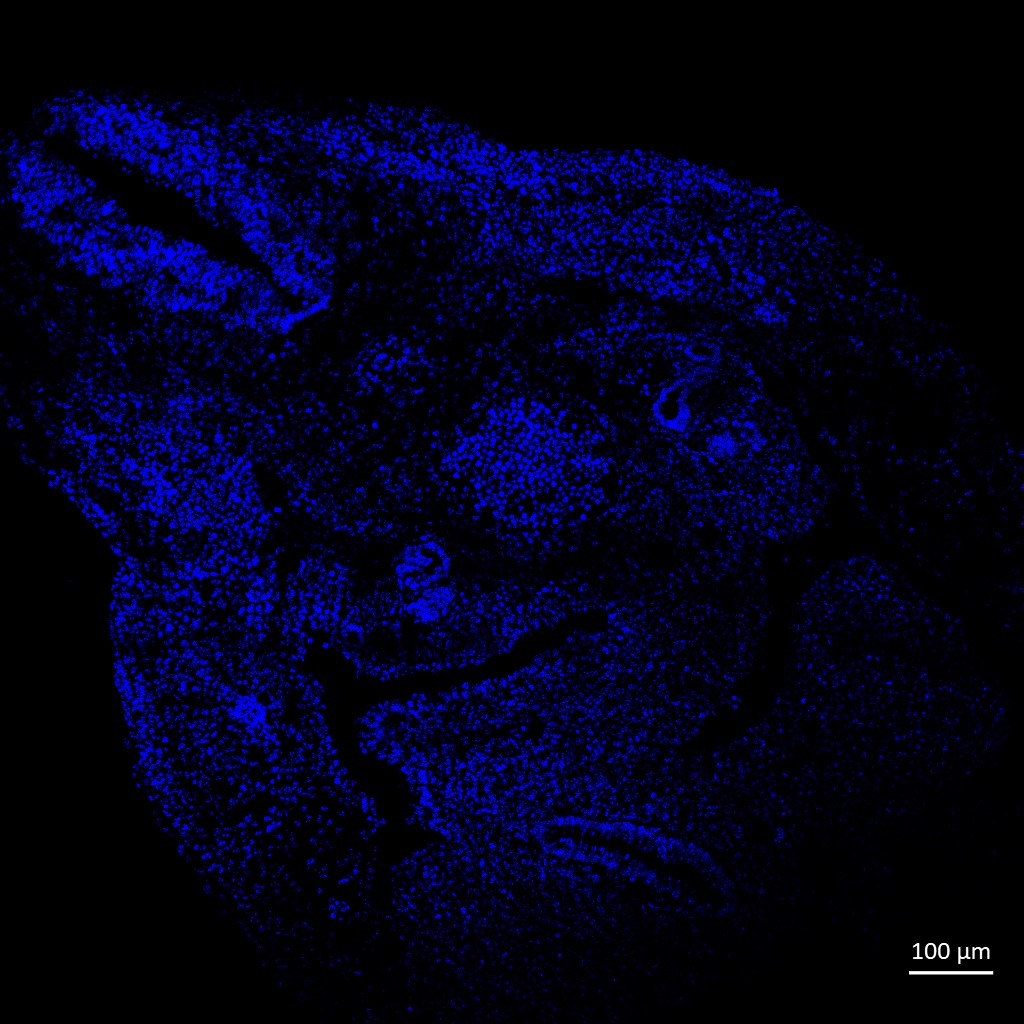

Supplement: Supplementary file 6 — Source data Fig. 1 [file 44318_2024_79_MOESM6_ESM.zip › source data-Figure 1/source data-Figure 1I/Figure I-3/Figure I-3_Hoechst.jpg]

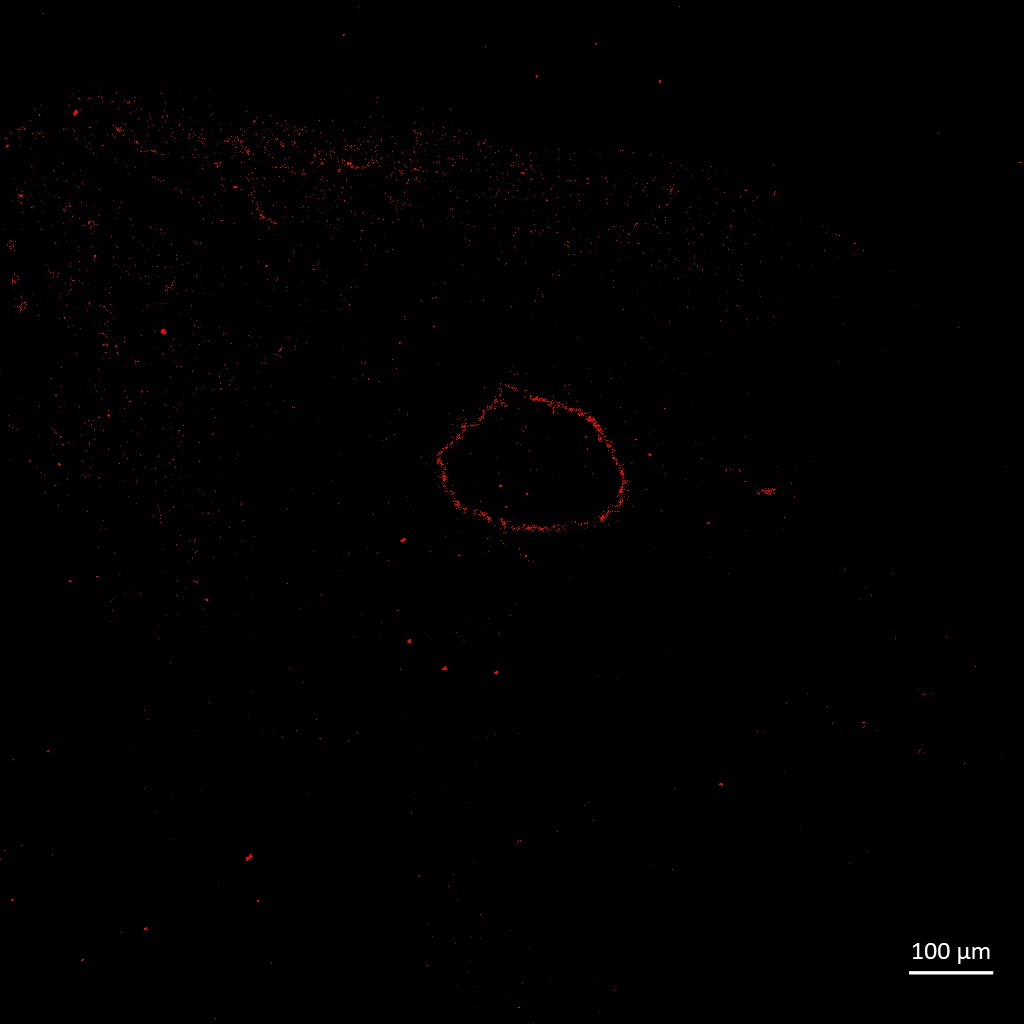

Supplement: Supplementary file 6 — Source data Fig. 1 [file 44318_2024_79_MOESM6_ESM.zip › source data-Figure 1/source data-Figure 1I/Figure I-3/Figure I-3_CD34.jpg]

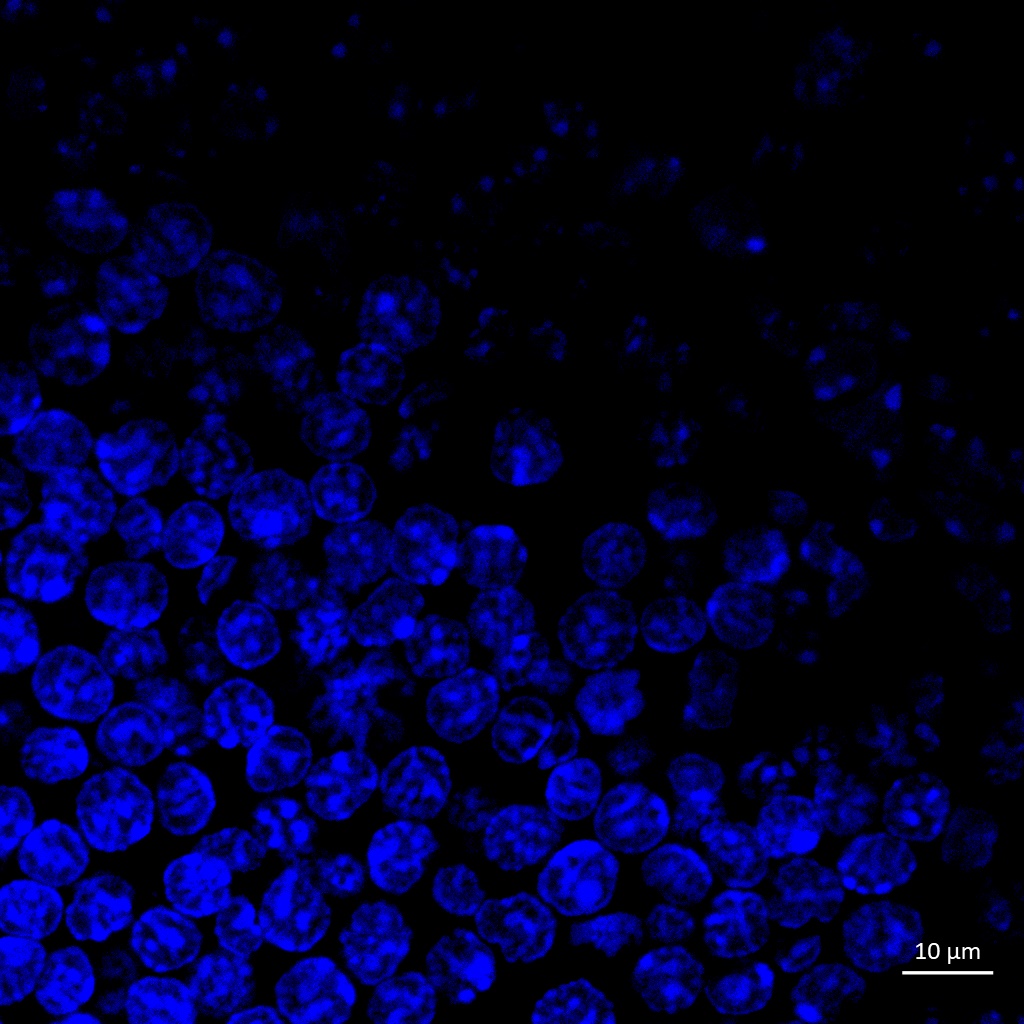

Supplement: Supplementary file 6 — Source data Fig. 1 [file 44318_2024_79_MOESM6_ESM.zip › source data-Figure 1/source data-Figure 1I/Figure I-4/Figure I-4_Hoechst.jpg]

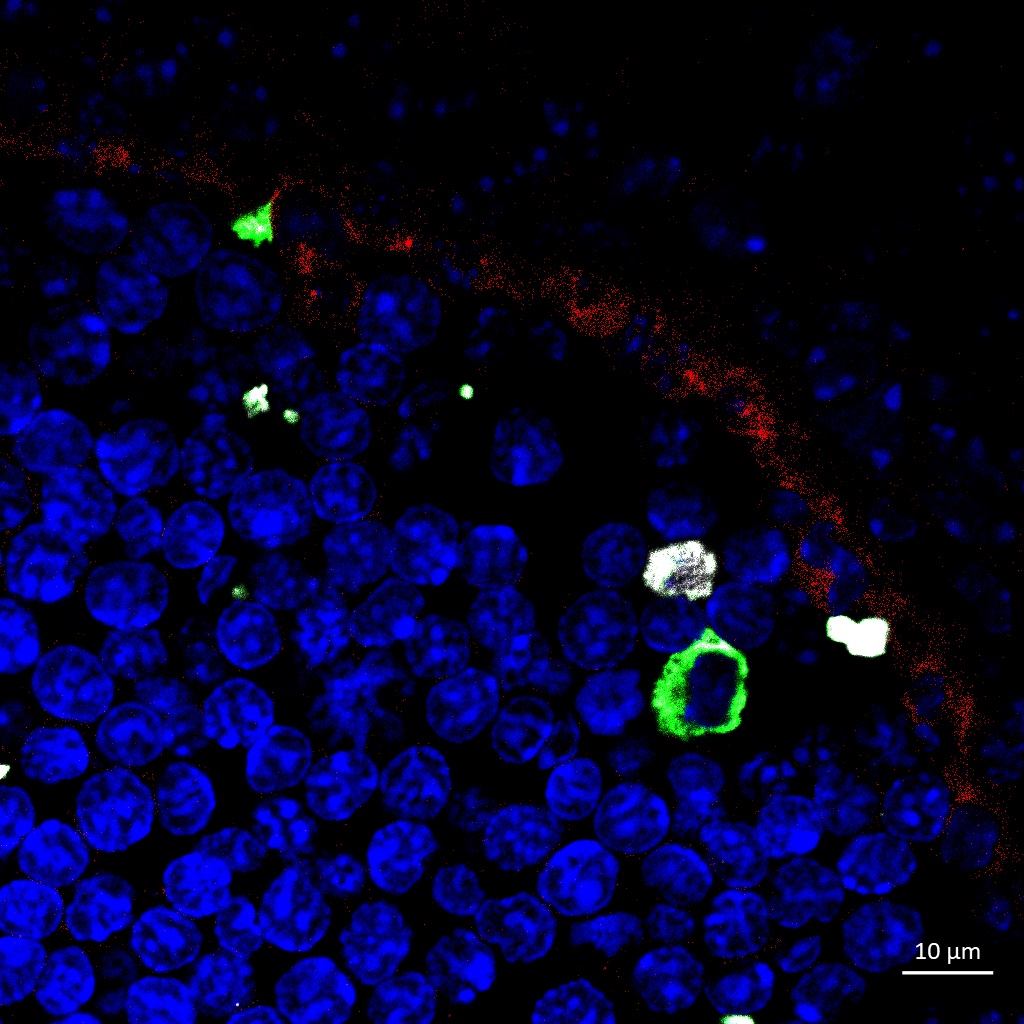

Supplement: Supplementary file 6 — Source data Fig. 1 [file 44318_2024_79_MOESM6_ESM.zip › source data-Figure 1/source data-Figure 1I/Figure I-4/Figure I-4_Merge.jpg]

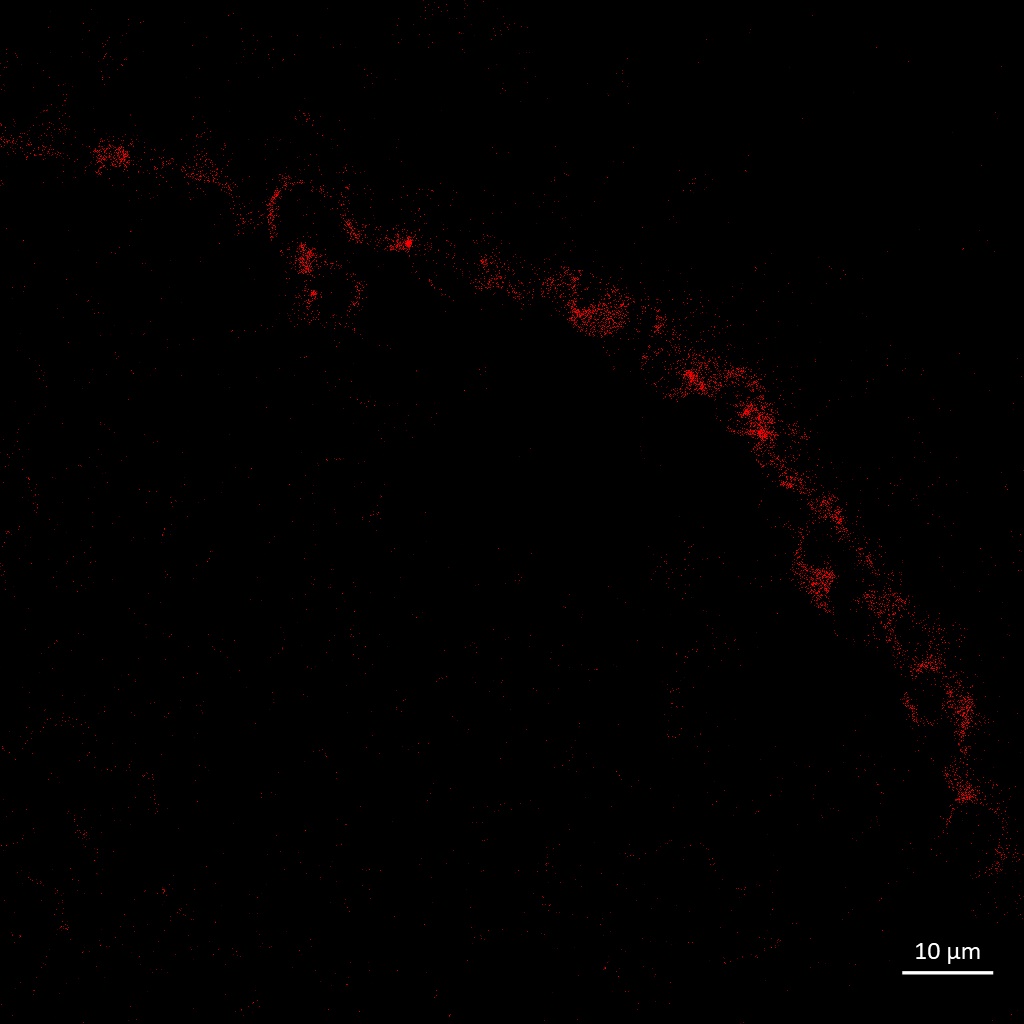

Supplement: Supplementary file 6 — Source data Fig. 1 [file 44318_2024_79_MOESM6_ESM.zip › source data-Figure 1/source data-Figure 1I/Figure I-4/Figure I-4_CD34.jpg]

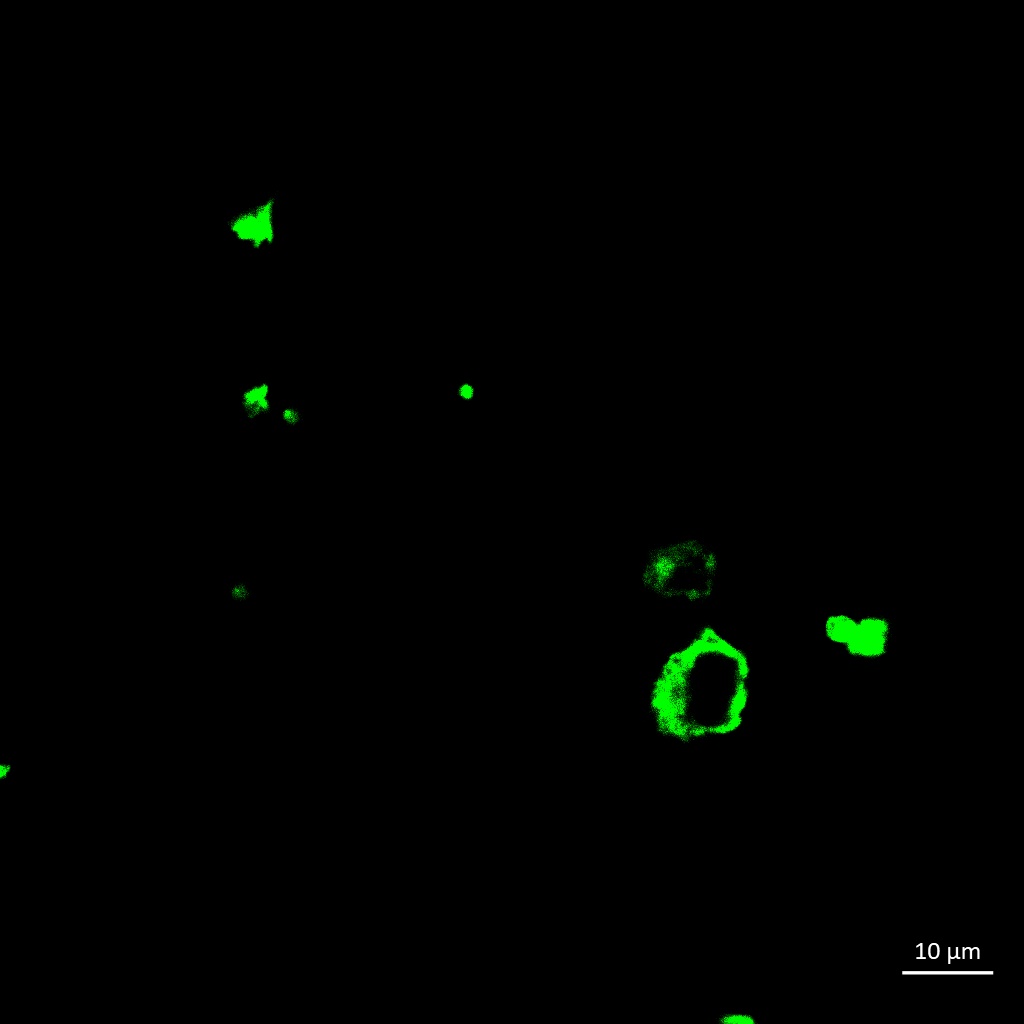

Supplement: Supplementary file 6 — Source data Fig. 1 [file 44318_2024_79_MOESM6_ESM.zip › source data-Figure 1/source data-Figure 1I/Figure I-4/Figure I-4_CD41.jpg]

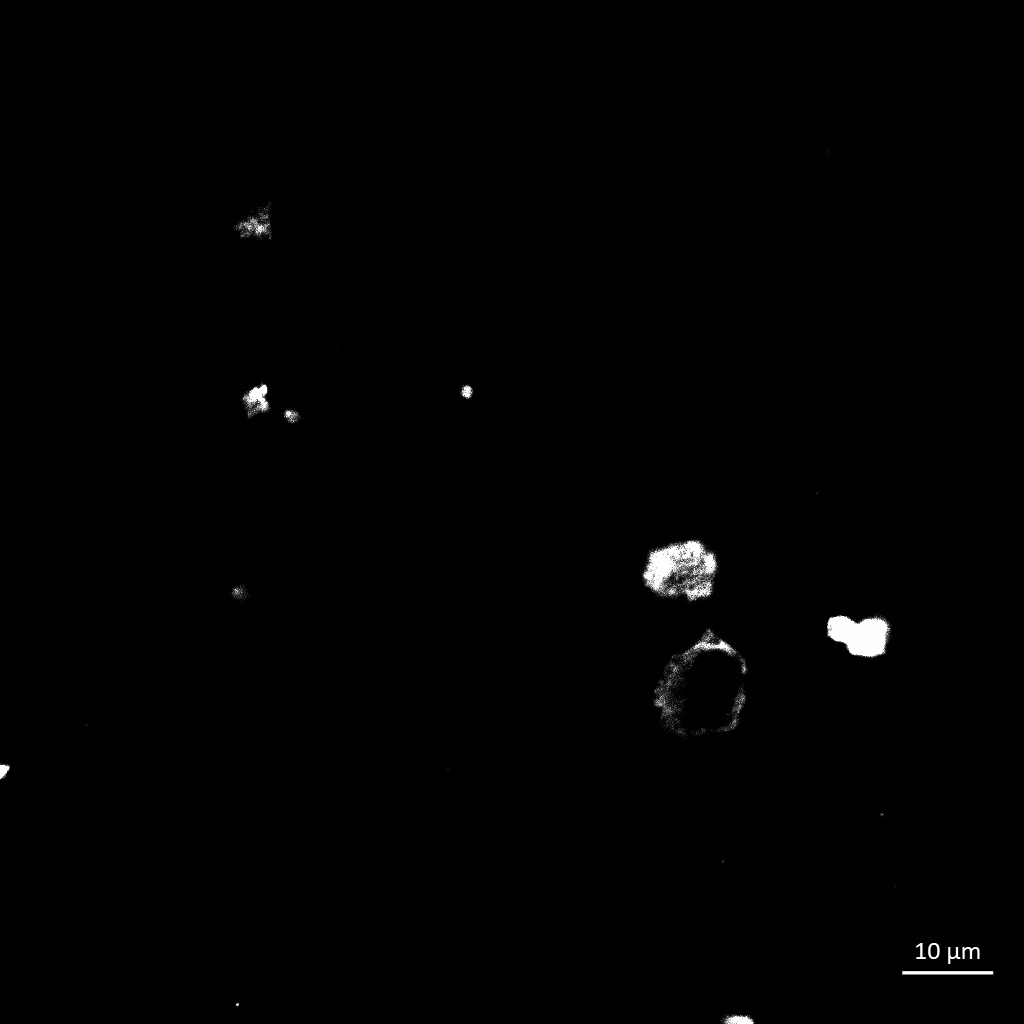

Supplement: Supplementary file 6 — Source data Fig. 1 [file 44318_2024_79_MOESM6_ESM.zip › source data-Figure 1/source data-Figure 1I/Figure I-4/Figure I-4_tdT.jpg]

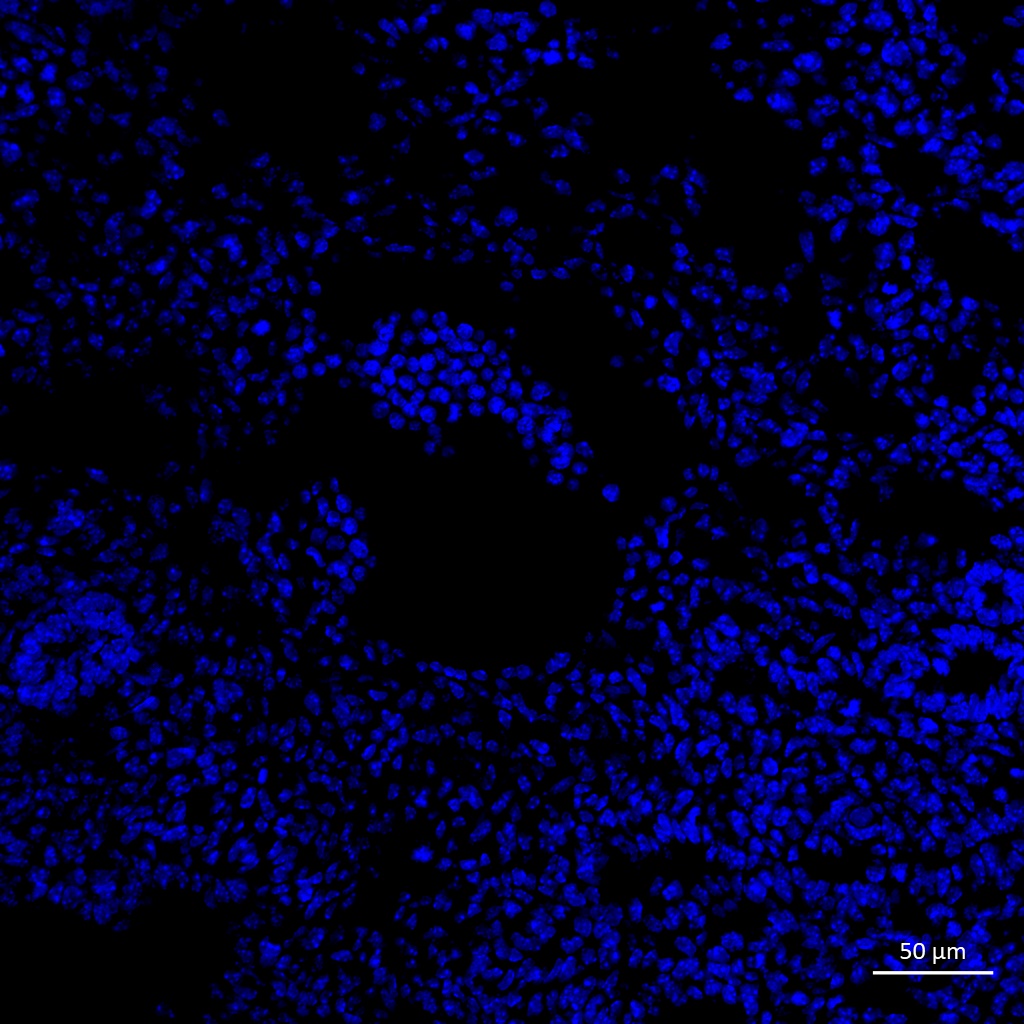

Supplement: Supplementary file 9 — Source data Fig. 4 [file 44318_2024_79_MOESM9_ESM.zip › source data-Figure 4/source data-Figure 4A/Figure 4A Ctr/Figure 4A Ctr_Hoechst.jpg]

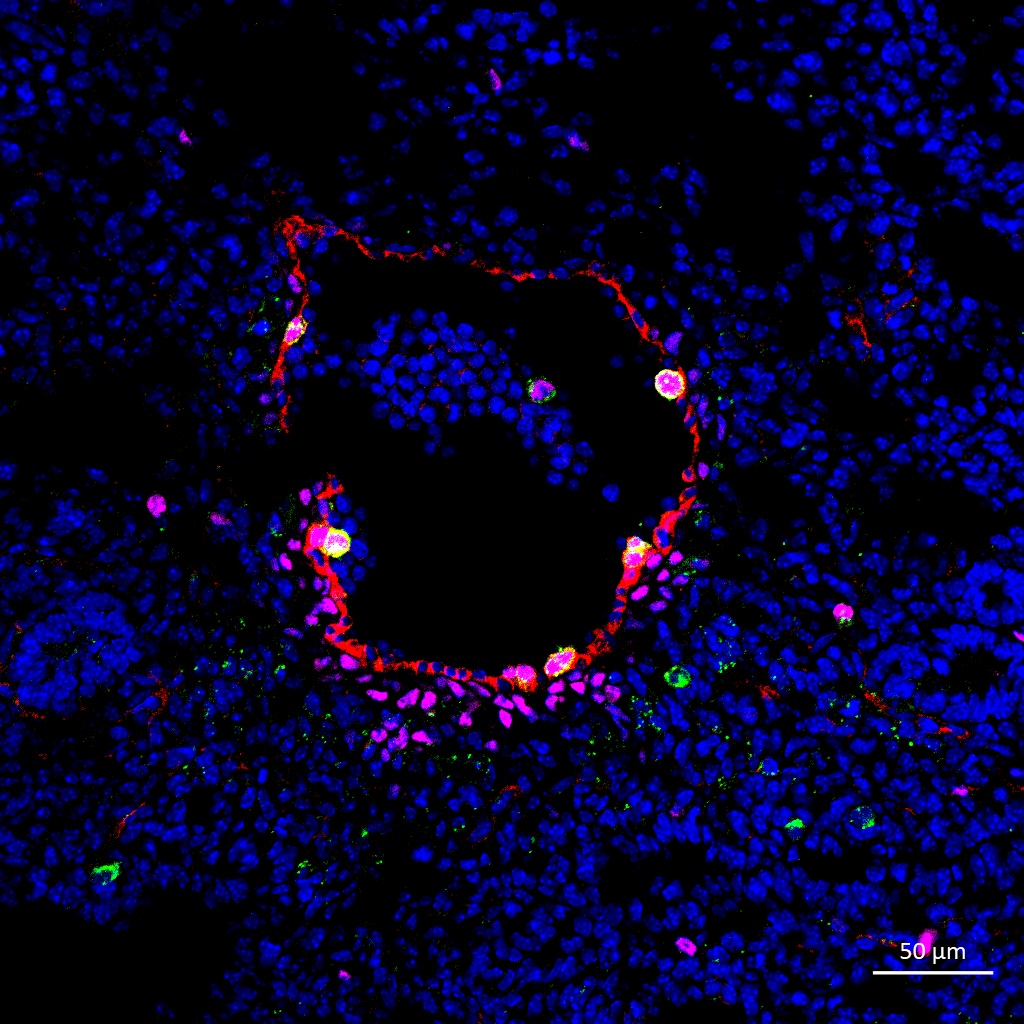

Supplement: Supplementary file 9 — Source data Fig. 4 [file 44318_2024_79_MOESM9_ESM.zip › source data-Figure 4/source data-Figure 4A/Figure 4A Ctr/Figure 4A Ctr_Merge.jpg]

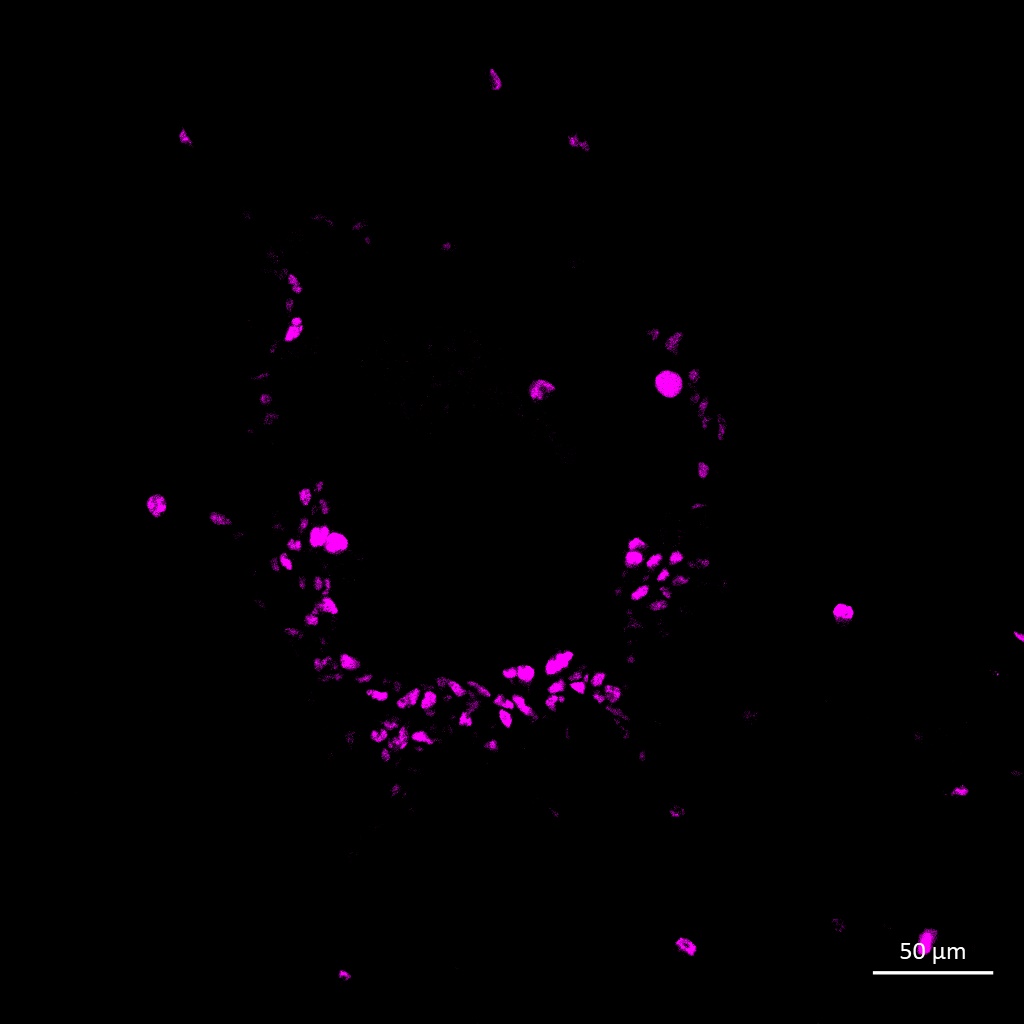

Supplement: Supplementary file 9 — Source data Fig. 4 [file 44318_2024_79_MOESM9_ESM.zip › source data-Figure 4/source data-Figure 4A/Figure 4A Ctr/Figure 4A Ctr_Runx1.jpg]

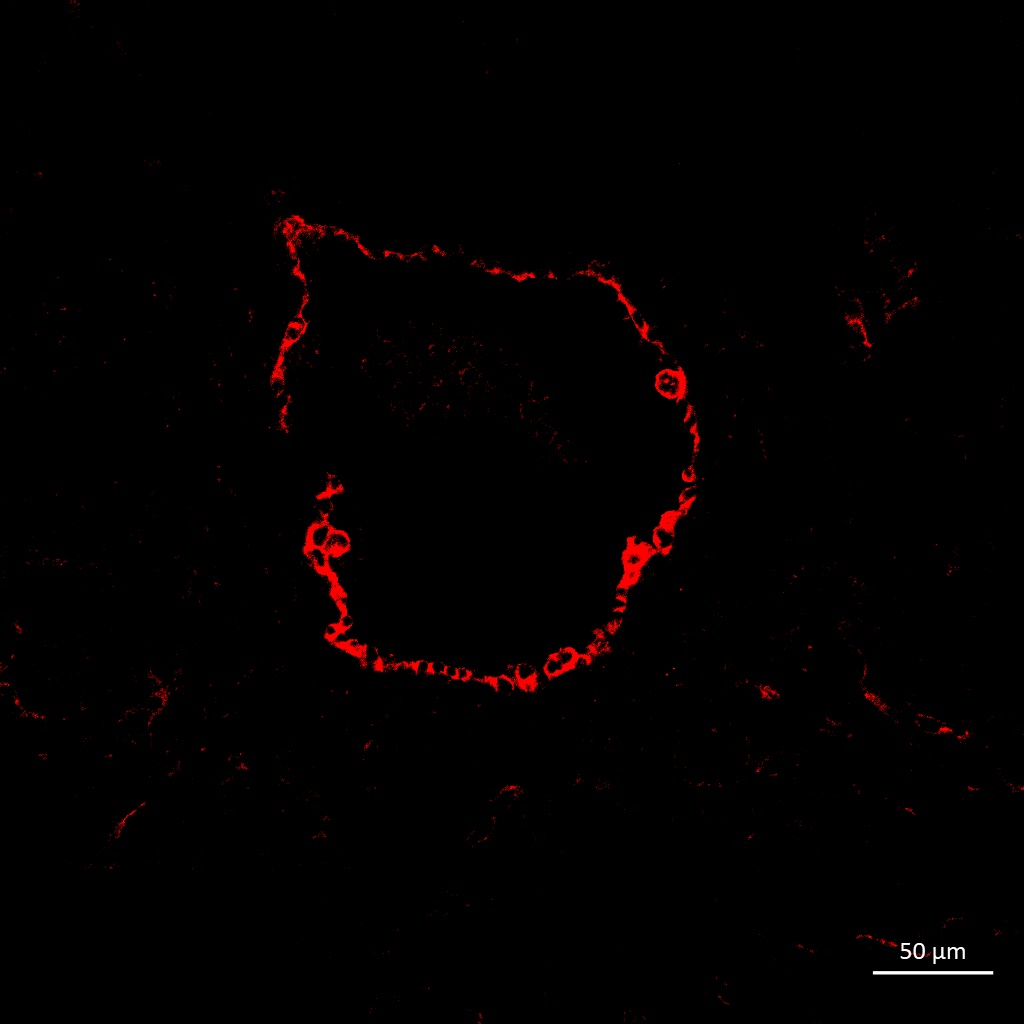

Supplement: Supplementary file 9 — Source data Fig. 4 [file 44318_2024_79_MOESM9_ESM.zip › source data-Figure 4/source data-Figure 4A/Figure 4A Ctr/Figure 4A Ctr_CD34.jpg]

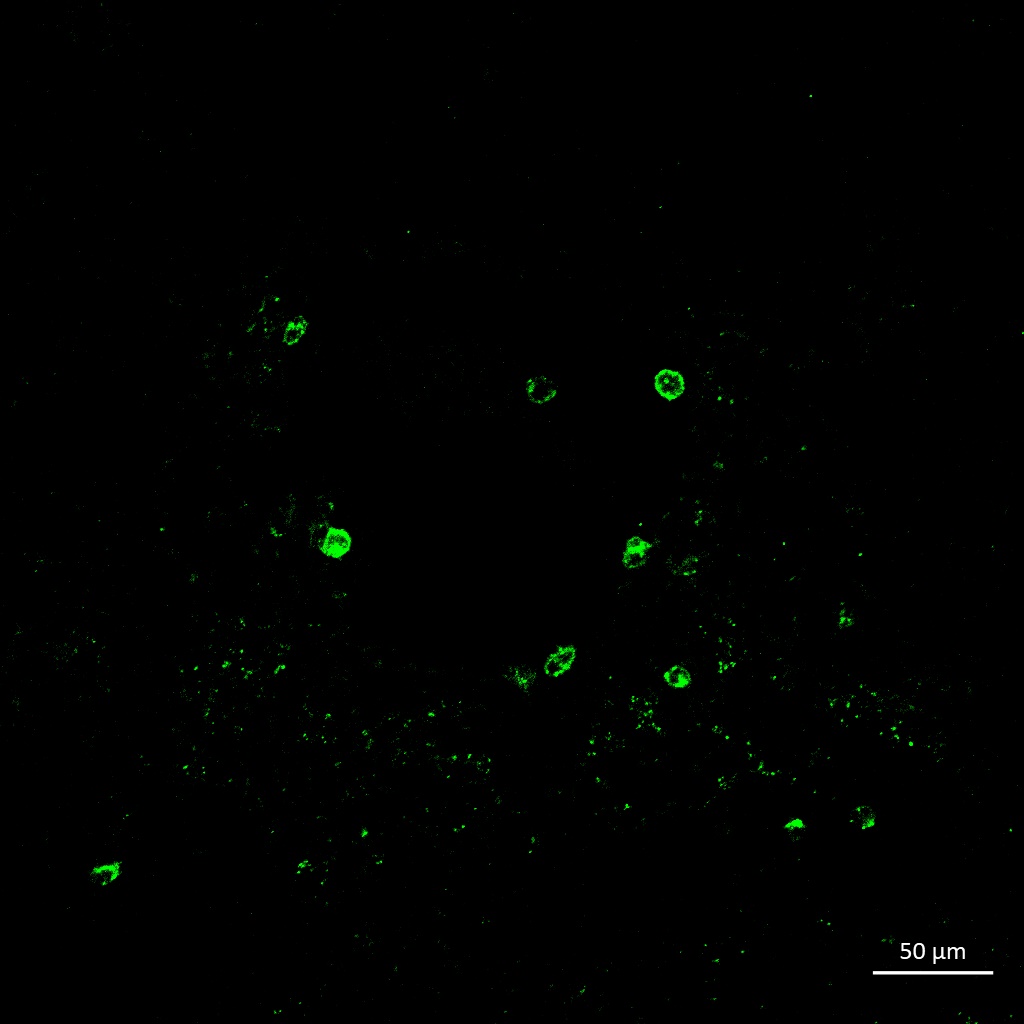

Supplement: Supplementary file 9 — Source data Fig. 4 [file 44318_2024_79_MOESM9_ESM.zip › source data-Figure 4/source data-Figure 4A/Figure 4A Ctr/Figure 4A Ctr_c-Kit.jpg]

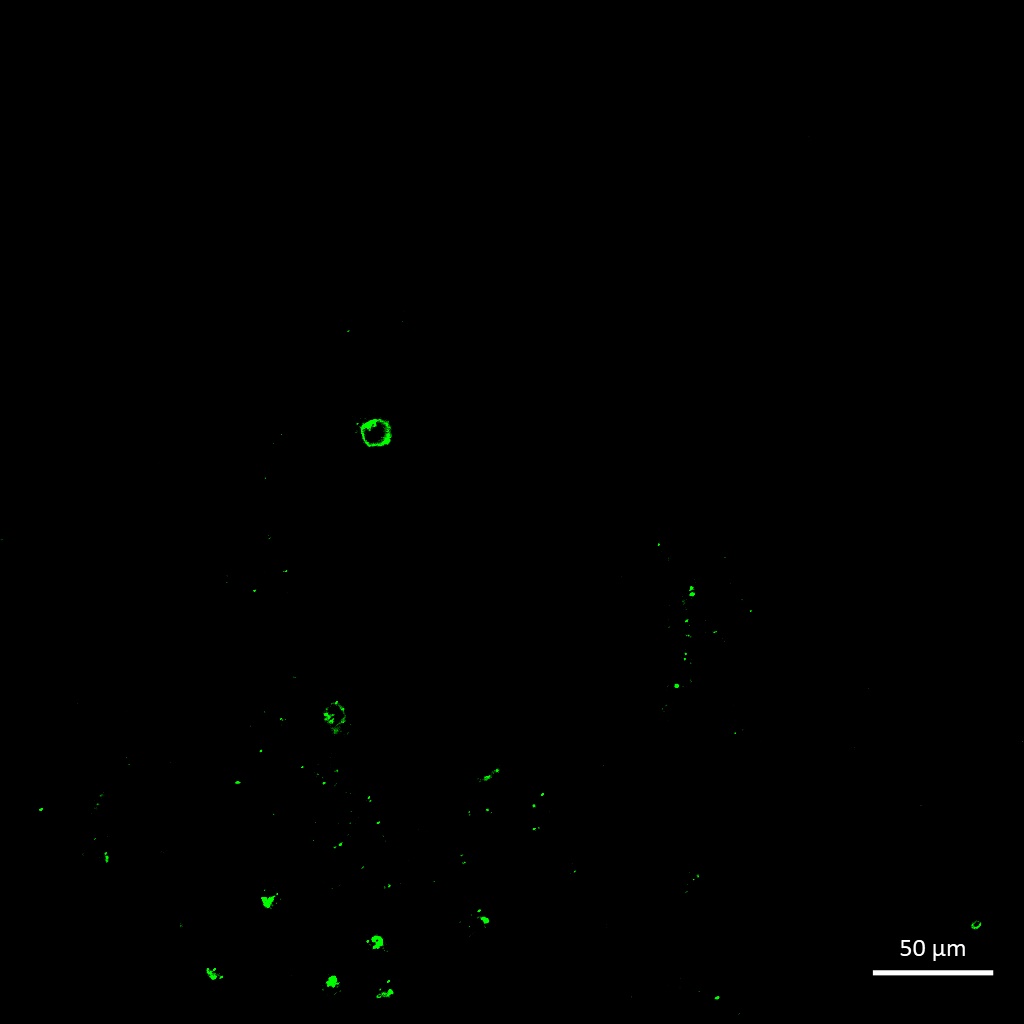

Supplement: Supplementary file 9 — Source data Fig. 4 [file 44318_2024_79_MOESM9_ESM.zip › source data-Figure 4/source data-Figure 4A/Figure 4A DTA/Figure 4A DTA_c-Kit.jpg]

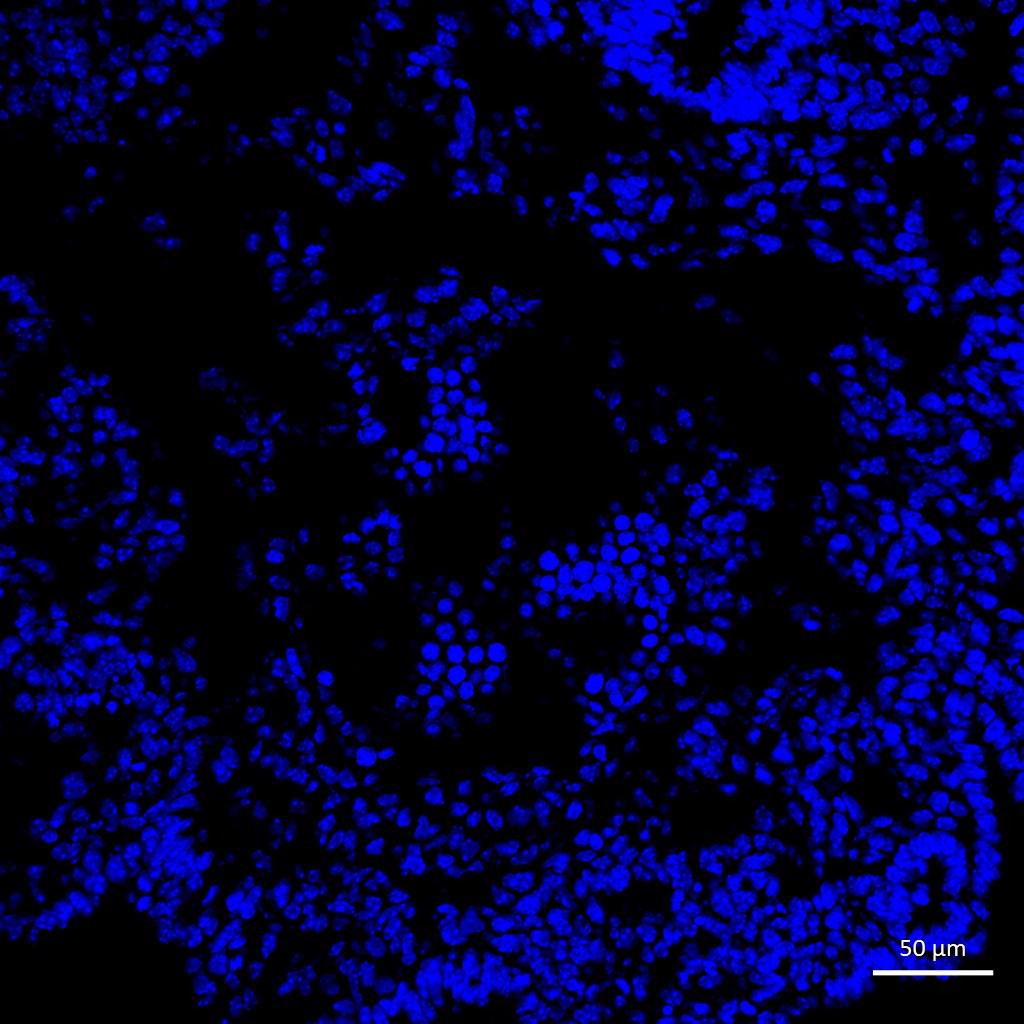

Supplement: Supplementary file 9 — Source data Fig. 4 [file 44318_2024_79_MOESM9_ESM.zip › source data-Figure 4/source data-Figure 4A/Figure 4A DTA/Figure 4A DTA_Hoechst.jpg]

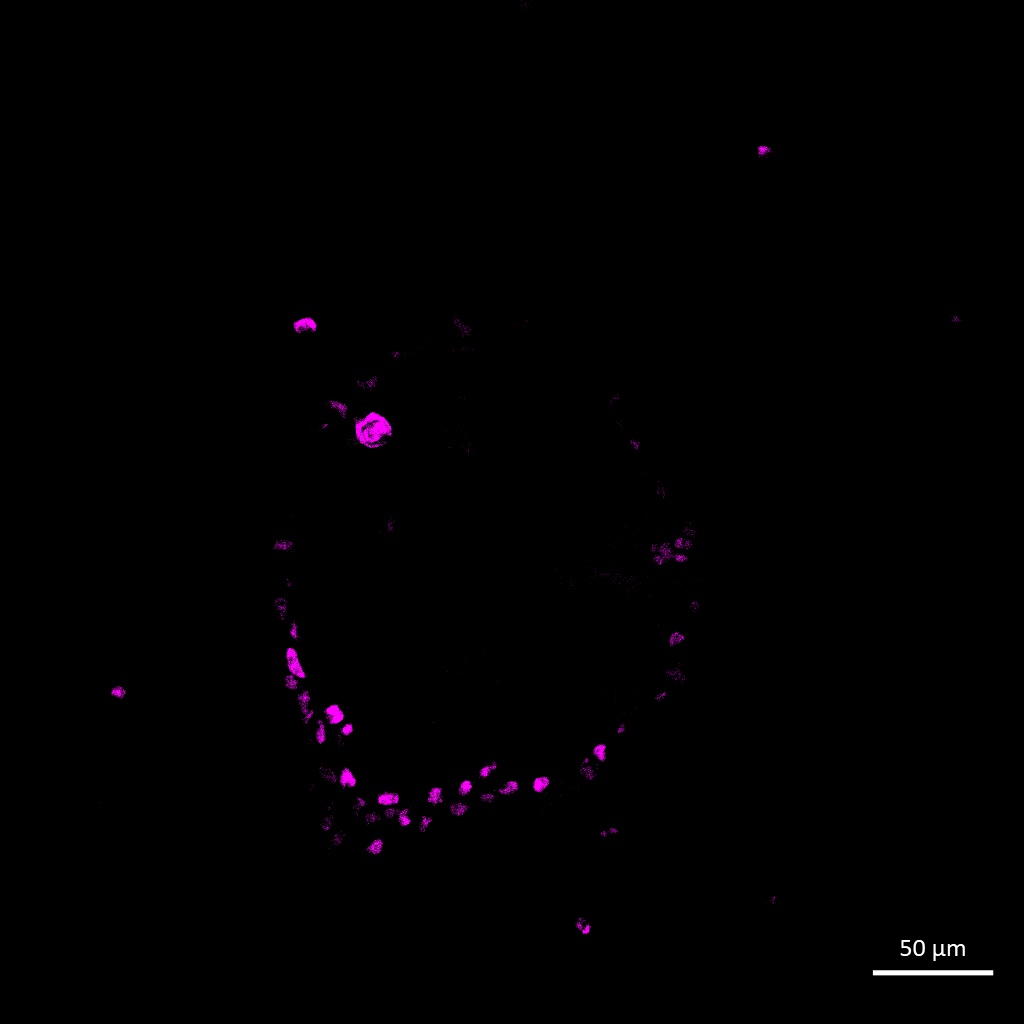

Supplement: Supplementary file 9 — Source data Fig. 4 [file 44318_2024_79_MOESM9_ESM.zip › source data-Figure 4/source data-Figure 4A/Figure 4A DTA/Figure 4A DTA_Runx1.jpg]

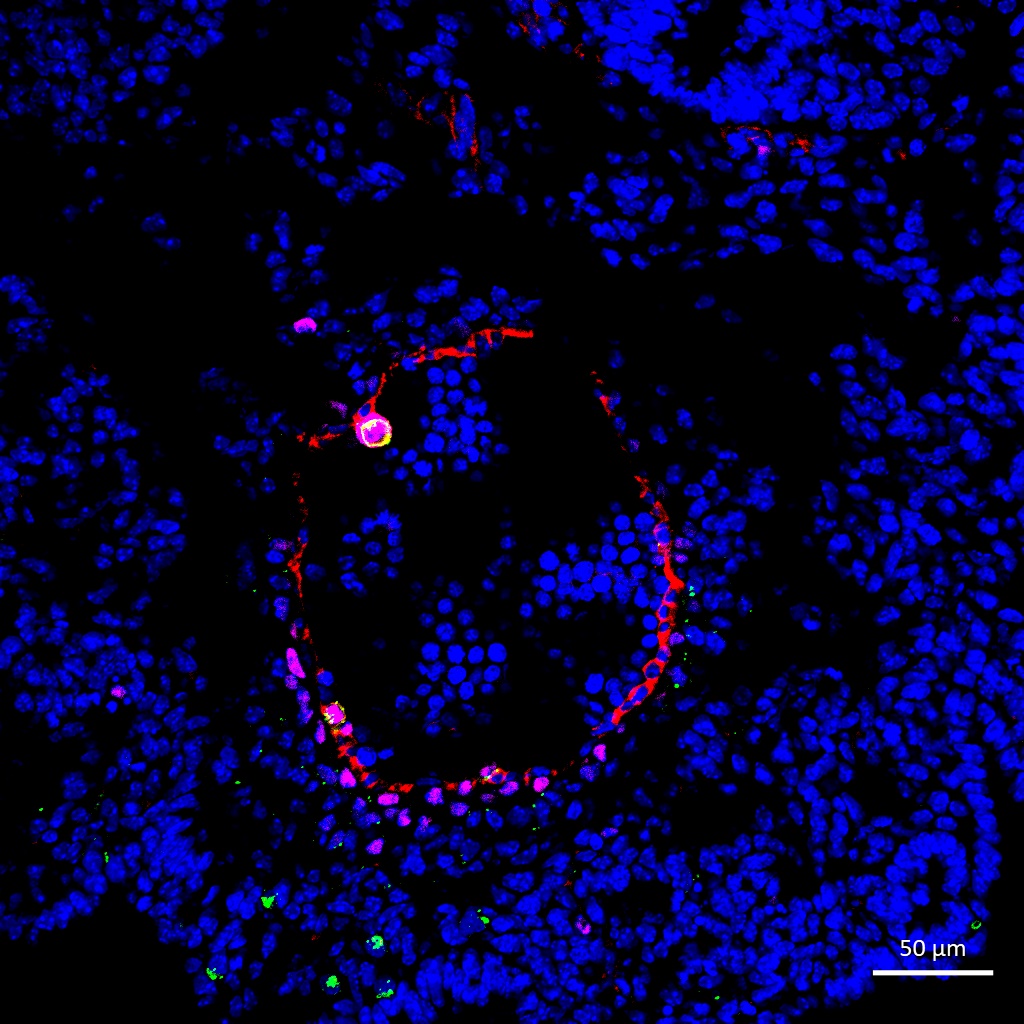

Supplement: Supplementary file 9 — Source data Fig. 4 [file 44318_2024_79_MOESM9_ESM.zip › source data-Figure 4/source data-Figure 4A/Figure 4A DTA/Figure 4A DTA_Merge.jpg]

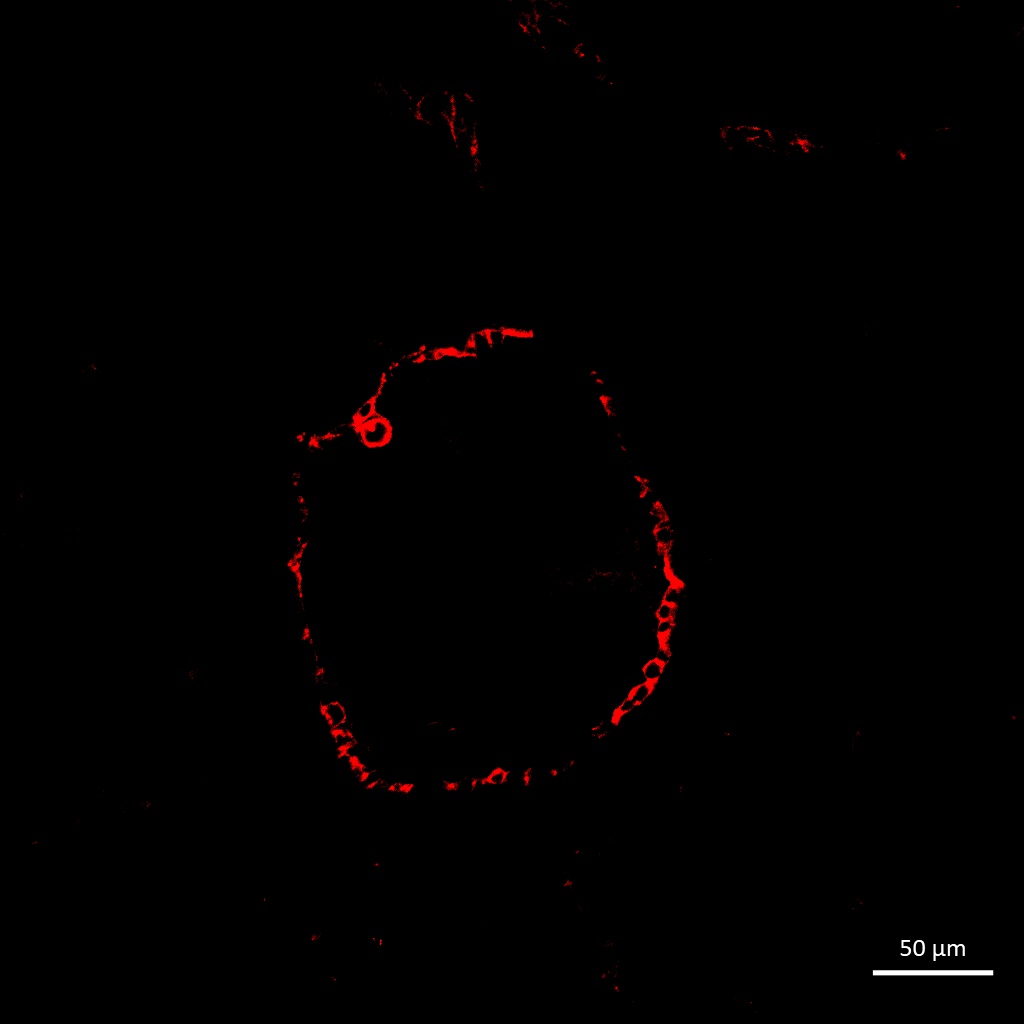

Supplement: Supplementary file 9 — Source data Fig. 4 [file 44318_2024_79_MOESM9_ESM.zip › source data-Figure 4/source data-Figure 4A/Figure 4A DTA/Figure 4A DTA_CD34.jpg]

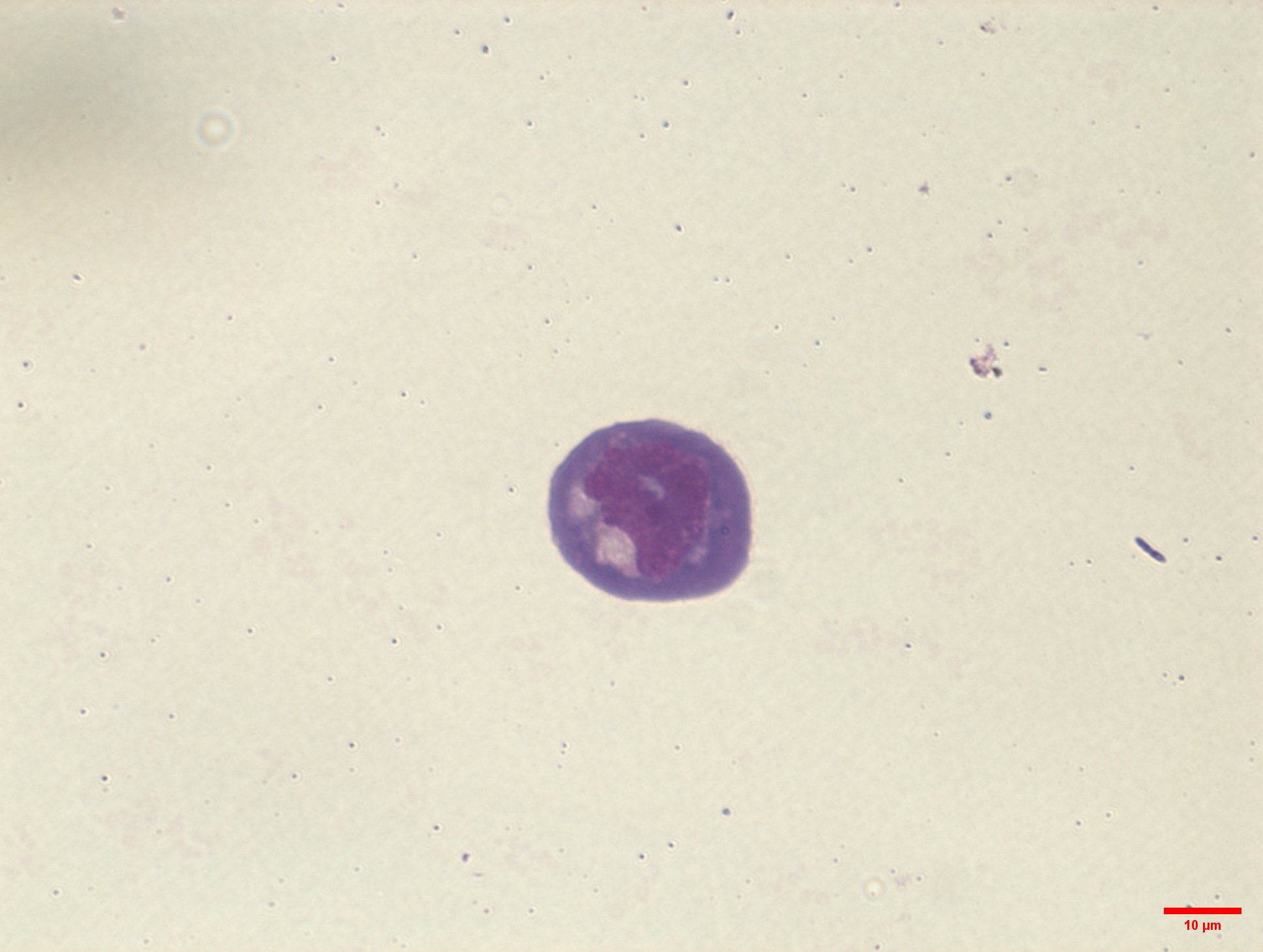

Supplement: Supplementary file 10 — Source data Fig. 6 [file 44318_2024_79_MOESM10_ESM.zip › source data-Figure 6/source data-Figure 6H/CD226- MK/CD226- MK-2.jpg]

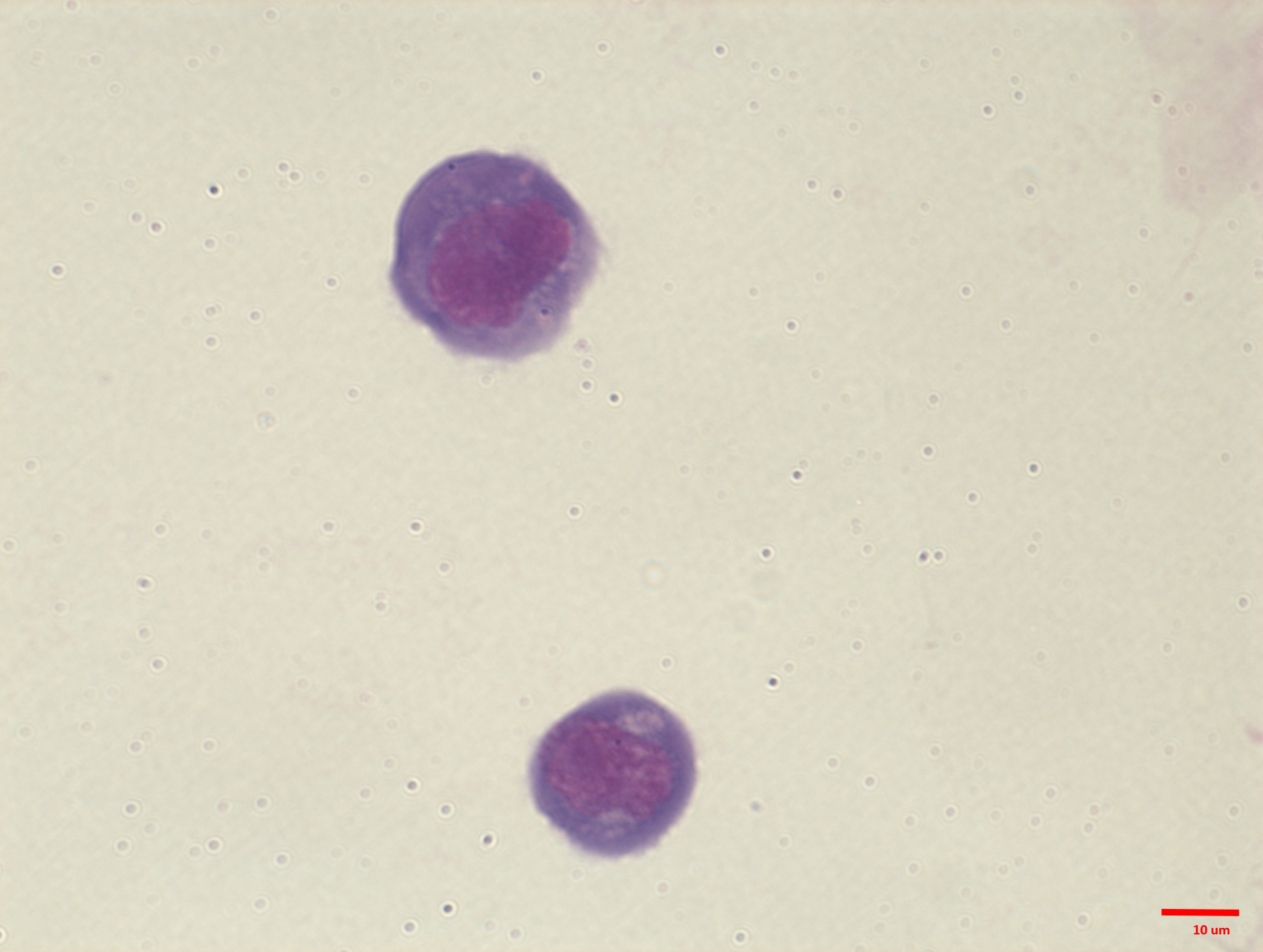

Supplement: Supplementary file 10 — Source data Fig. 6 [file 44318_2024_79_MOESM10_ESM.zip › source data-Figure 6/source data-Figure 6H/CD226- MK/CD226- MK-3.jpg]

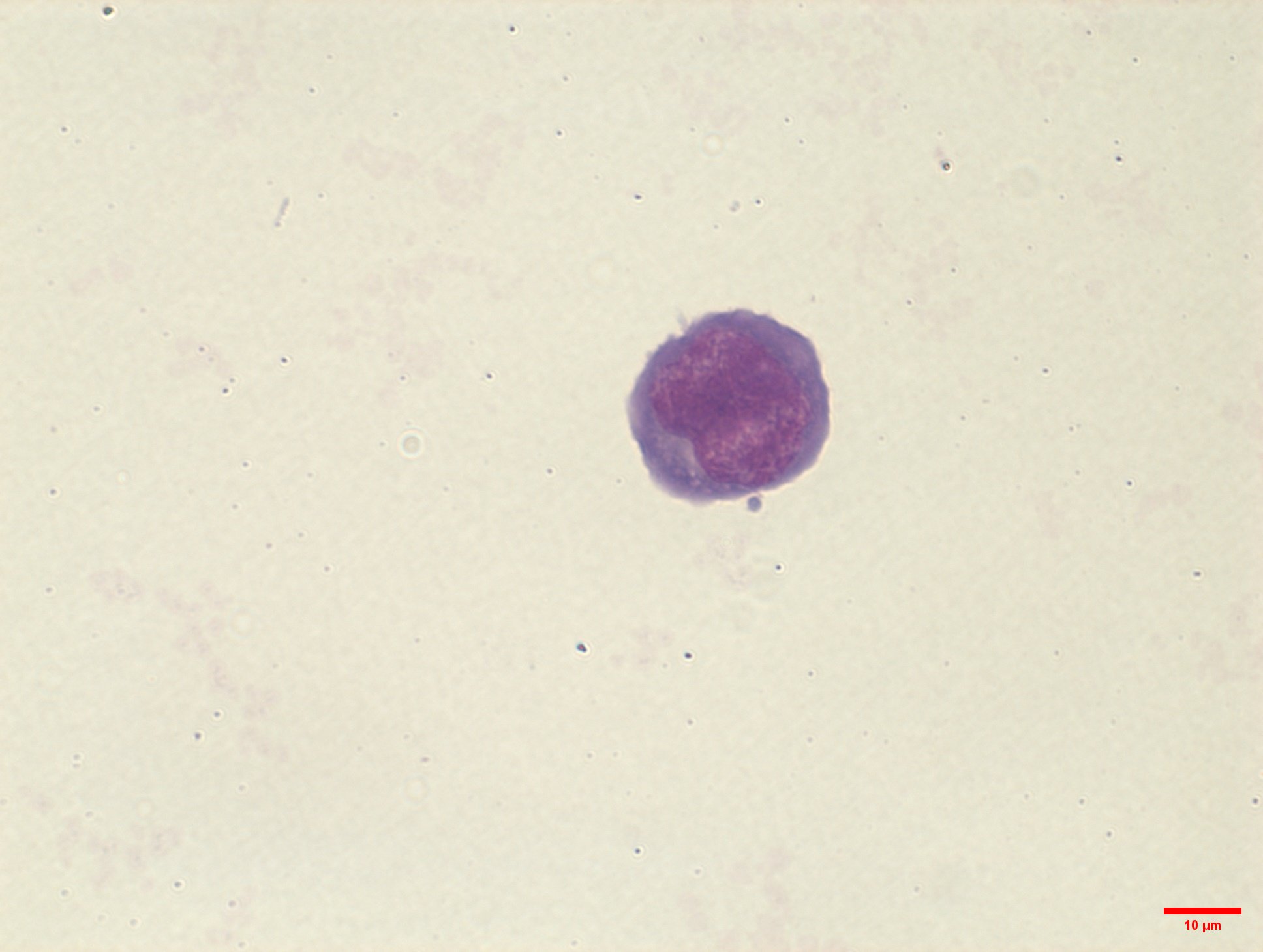

Supplement: Supplementary file 10 — Source data Fig. 6 [file 44318_2024_79_MOESM10_ESM.zip › source data-Figure 6/source data-Figure 6H/CD226- MK/CD226- MK-1.jpg]

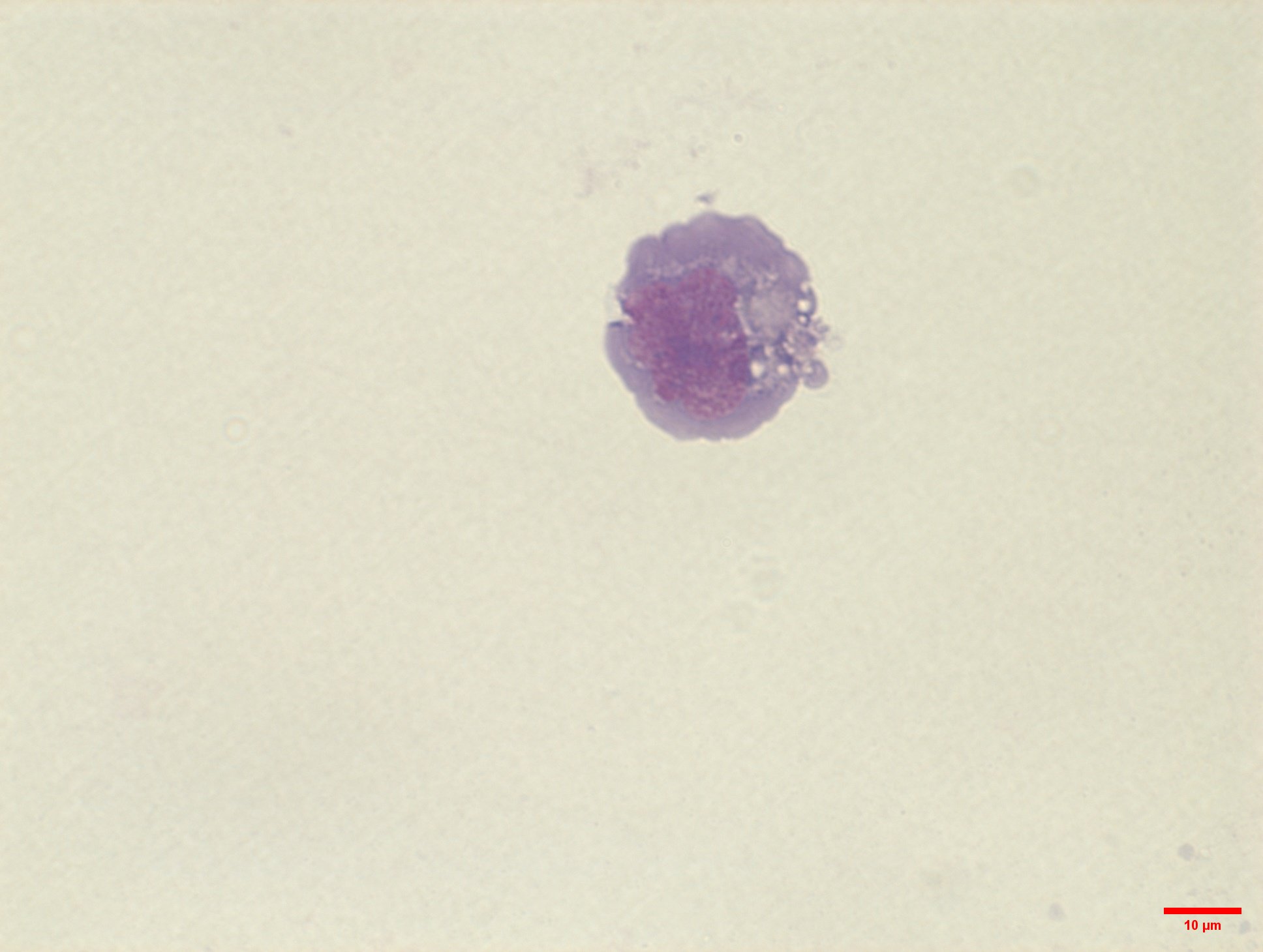

Supplement: Supplementary file 10 — Source data Fig. 6 [file 44318_2024_79_MOESM10_ESM.zip › source data-Figure 6/source data-Figure 6H/CD226+ MK/CD226+ MK-2.jpg]

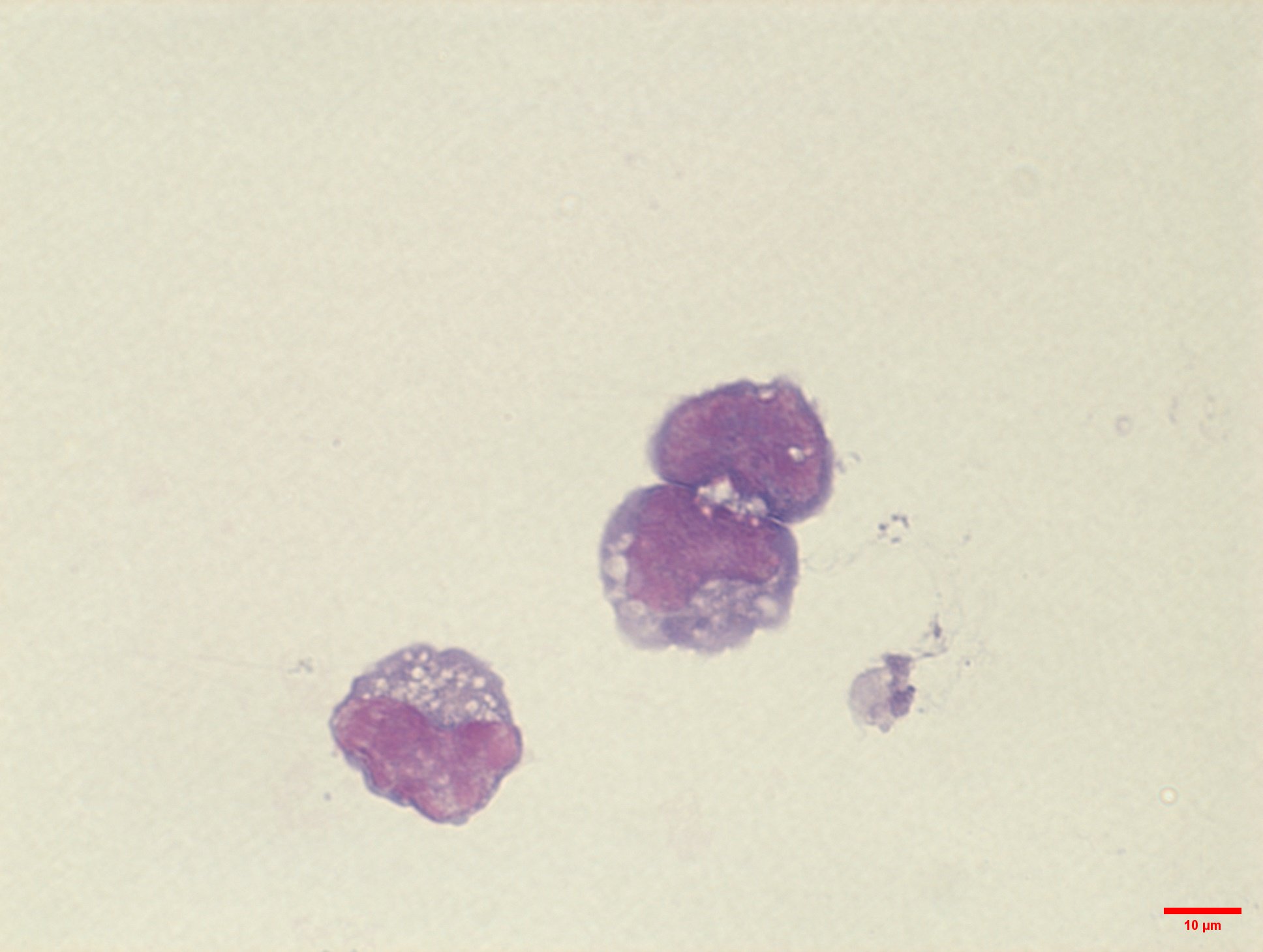

Supplement: Supplementary file 10 — Source data Fig. 6 [file 44318_2024_79_MOESM10_ESM.zip › source data-Figure 6/source data-Figure 6H/CD226+ MK/CD226+ MK-3.jpg]

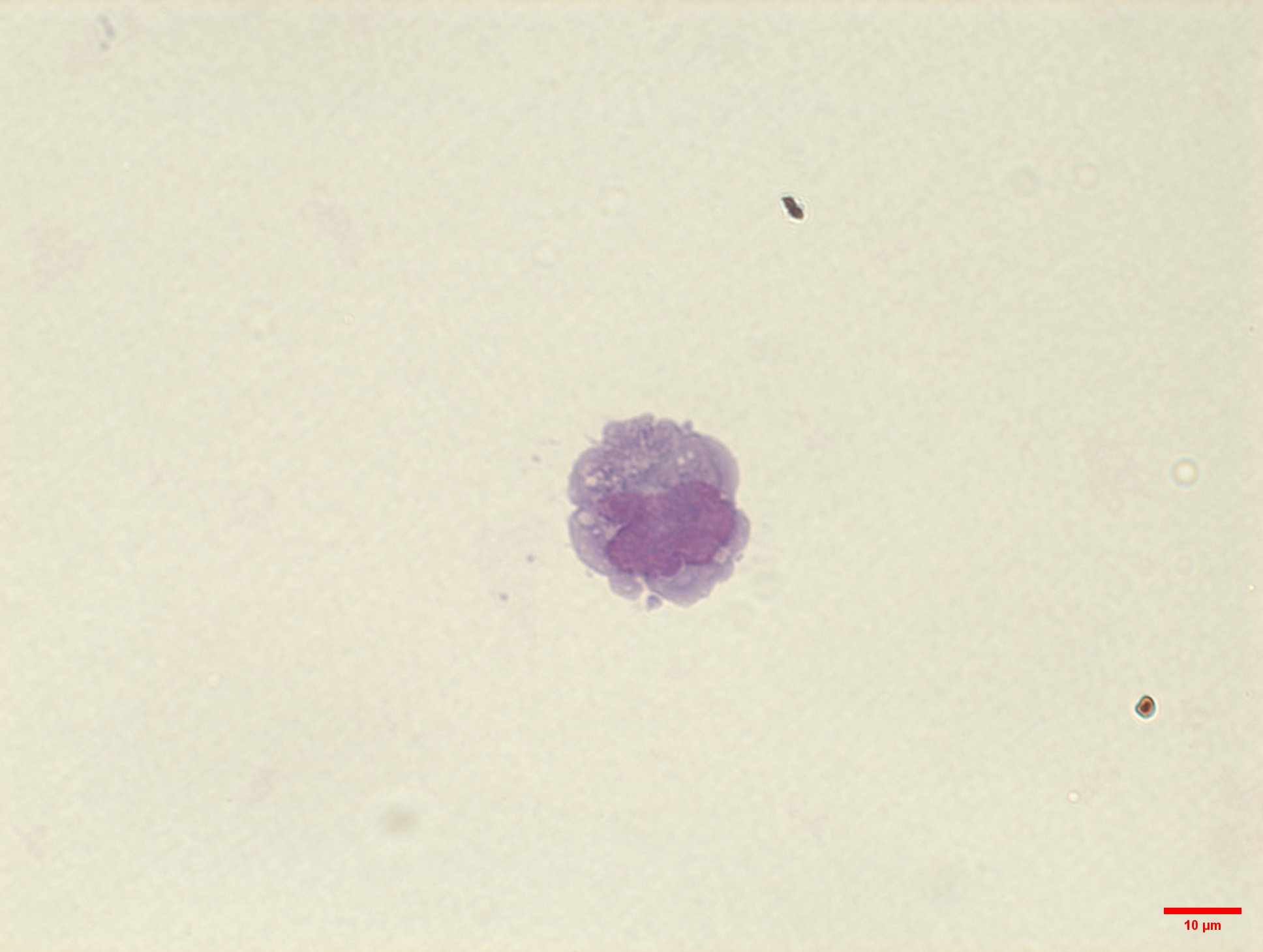

Supplement: Supplementary file 10 — Source data Fig. 6 [file 44318_2024_79_MOESM10_ESM.zip › source data-Figure 6/source data-Figure 6H/CD226+ MK/CD226+ MK-1.jpg]
